# Development of a chemical scaffold for inhibiting nonribosomal peptide synthetases in live bacterial cells

**DOI:** 10.3762/bjoc.20.39

**Published:** 2024-02-26

**Authors:** Fumihiro Ishikawa, Sho Konno, Hideaki Kakeya, Genzoh Tanabe

**Affiliations:** 1 Faculty of Pharmacy, Kindai University, 3-4-1 Kowakae, Higashi-Osaka, Osaka 577-8502, Japan; 2 Department of System Chemotherapy and Molecular Sciences, Division of Medicinal Frontier Sciences, Graduate School of Pharmaceutical Sciences, Kyoto University, Sakyo, Kyoto 606-8501, Japan

**Keywords:** adenylation domain inhibitor, gramicidin S synthetase, natural product, nonribosomal peptide, nonribosomal peptide synthetase

## Abstract

The adenylation (A) domain is essential for non-ribosomal peptide synthetases (NRPSs), which synthesize various peptide-based natural products, including virulence factors, such as siderophores and genotoxins. Hence, the inhibition of A-domains could attenuate the virulence of pathogens. 5’-*O*-*N*-(Aminoacyl or arylacyl)sulfamoyladenosine (AA-AMS) is a bisubstrate small-molecule inhibitor of the A-domains of NRPSs. However, the bacterial cell permeability of AA-AMS is typically a problem owing to its high hydrophilicity. In this study, we investigated the influence of a modification of 2′-OH in the AMS scaffold with different functional groups on binding to target enzymes and bacterial cell penetration. The inhibitor **7** with a cyanomethyl group at 2′-OH showed desirable inhibitory activity against both recombinant and intracellular gramicidin S synthetase A (GrsA) in the gramicidin S-producer *Aneurinibacillus migulanus* ATCC 9999, providing an alternative scaffold to develop novel A-domain inhibitors.

## Introduction

**Figure 1:**
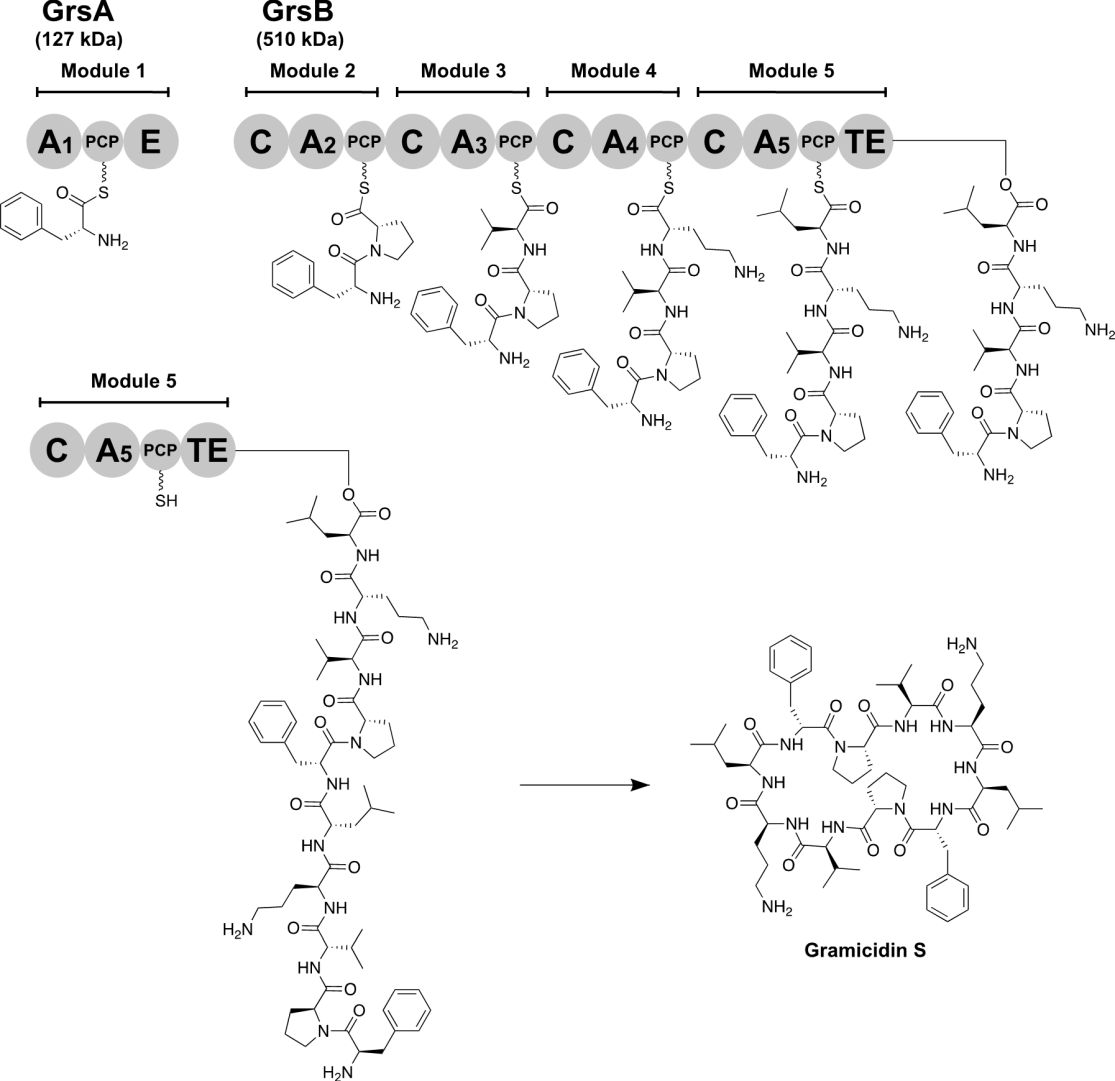
Biosynthesis of gramicidin S. Modules comprise the PCP, A, E, C, and TE domains. PCP, peptidyl carrier protein; A1, ʟ-Phe-specific A-domain; A2, ʟ-Pro-specific A-domain; A3, ʟ-Val-specific A-domain; A4, ʟ-Orn-specific A-domain; A5, ʟ-Leu-specific A-domain; E, epimerization domain; C, condensation domain; TE, thioesterase domain.

**Figure 2:**
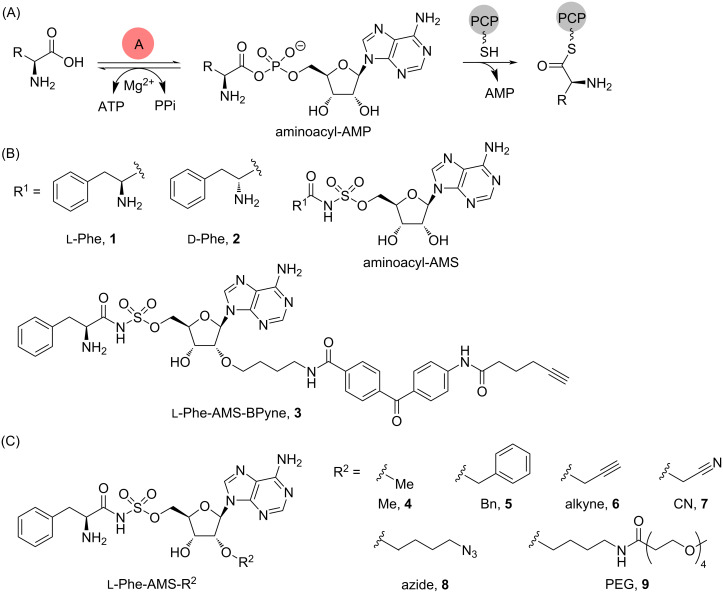
(A) Adenylation reaction in a nonribosomal peptide synthetase. (B) Structures of aminoacyl-AMS inhibitors and the ʟ-Phe-AMS-BPyne probe. (C) Structures of 2′-OH-substituted ʟ-Phe-AMS derivatives for A-domains. PCP, peptidyl carrier protein; AMS, 5′-*O*-sulfamoyladenosine.

Nonribosomal peptides (NRPs) exhibit various biological activities and have been used as therapeutic agents, such as antibiotics, anticancer agents, and immunosuppressants [1]. Additionally, NRPs function as virulence factors, such as siderophores and genotoxins [2]. Therefore, inhibiting their biosynthesis by using small molecules can help to elucidate their natural functions and their potential as therapeutic targets. NRPs are synthesized by large, versatile, and multifunctional proteins called nonribosomal peptide synthetases (NRPSs), which are composed of multiple modules and subdivided domains (Figure 1) [3]. The adenylation (A) domain in NRPSs is responsible for the selection and activation of amino acids, hydroxy acids, and aryl acids upon ATP consumption (Figure 2a) [4]. The activated aminoacyladenosine monophosphate (AMP) is transferred to the thiol group of a phosphopantetheine prosthetic arm in an adjacent peptidyl carrier protein (PCP). The amino acid loaded on the PCP then undergoes coupling with the amino acid loaded on the downstream PCP in the condensation (C) domain. Finally, the linear peptide on the PCP in the last module is either hydrolyzed or cyclized by a thioesterase (TE) domain, consequently resulting in the formation of the final products.

Inhibitors that target each domain of NRPSs are valuable for elucidating the biosynthetic pathways associated with bioactive NRPs and for developing antibiotic molecules. Burkart et al. reported a systematic strategy for inhibiting modular synthases [5]. They used the inhibitors of individual domains to investigate the biosynthetic pathway of blue pigment synthetase A, which produces the blue pigment indigoidine, and demonstrated that their results complement the proposed biosynthetic pathway. Furthermore, among the catalytic domain inhibitors of NRPSs, A-domain inhibitors have been widely developed as potential therapeutic agents for treating infectious diseases.

Aryl acid A-domains are involved in the synthesis of several bacterial siderophores such as vibriobactin from *Vibrio cholera*, yersiniabactin from *Yersinia pestis*, and mycobactin from *Mycobacterium tuberculosis* [6]. 5′-*O*-Sulfamoyladenosine (AMS), a bioisosteric analog of an AMP intermediate, has been used as a non-hydrolysable scaffold for developing A-domain inhibitors. Moreover, 5′-*O*-[*N*-(salicyl)sulfamoyl]adenosine (Sal-AMS) and its derivatives show potent inhibitory activities against the A-domain of MbtA, a component of mycobactin synthetase and antimicrobial activities against *M*. *tuberculosis* [7]. In addition, aminoacyl (AA)-AMS has been designed to inhibit the amino acid-activating A-domains in NRPSs and has been found to be a tight-binding inhibitor (Figure 2b) [8]. Moreover, the intracellular concentrations of a series of AMS derivatives in *Escherichia coli*, *Bacillus subtilis*, and *Mycobacterium smegmatis* have been investigated by Tan et al., demonstrating non-obvious correlations between the chemical structure and permeability among various bacteria, owing to the differences in the composition of cell membranes and presence of efflux pumps [9].

**Figure 3:**
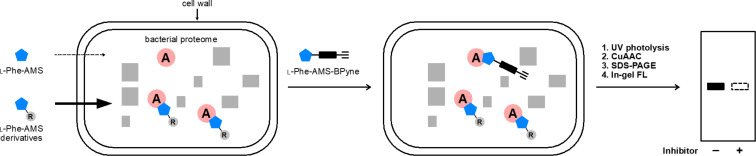
Illustration of the inhibition of A-domains by aminoacyl-AMS derivatives in live bacterial cells. Cell-permeable ʟ-Phe-AMS derivatives can penetrate cells and interact with A-domains in live bacterial cells, resulting in the competitive inhibition of the labeling by ʟ-Phe-AMS-BPyne. The substituent group (R; gray) of ʟ-Phe-AMS derivatives could facilitate their cell penetration. After UV irradiation (365 nm), the labeled proteins are treated with a TAMRA-N_3_ under copper(I)-catalyzed azide–alkyne cycloaddition conditions, followed by SDS-PAGE coupled with in-gel fluorescence scanning. AMS, 5′-*O*-sulfamoyladenosine.

Specific protein labeling using a chemical probe can help to identify, characterize, and visualize target proteins [10–11]. The first chemical probe used for A-domains in NRPSs was reported by Marahiel et al. [8]. They introduced a pegylated biotin linker at the 2′-OH group of ʟ-Phe-AMS and confirmed that the probe retains the binding activities toward the A-domain of GrsA, a gramicidin S synthetase. Aldrich et al. developed a Sal-AMS-based activity-based probe (ABP) to profile MbtA in *M. tuberculosis* [12]. In contrast, we previously described an activity-based protein profiling (ABPP) strategy for NRPSs using ABPs that target A-domains (Figure 2b) [13–15]. The probes comprise an aminoacyl-AMS ligand and a photoaffinity group with clickable alkyne functionality appended to the 2′-OH group of adenosine. A complex structure of the GrsA A-domain with ʟ-Phe and AMP revealed that the 2′-OH of the adenosine skeleton is oriented toward the outside of the active site of the GrsA A-domain, suggesting that chemical modification at the 2′-OH group of the adenosine skeleton would be tolerated [16] (Figure 2b). Moreover, these probes (AA-AMS-BPyne) can selectively label the A-domains corresponding to the amino acid of the ligand in both recombinant enzymes and proteomes. We recently reported that these probes can be used to label the A-domains of endogenous NRPSs in live bacterial cells [17–19]. The intracellular labeling of the enterobactin synthetase EntF with Sal-AMS-BPyne requires carbonyl cyanide *m*-chlorophenylhydrazone, which collapses the proton motive force used in most efflux pumps [17]. Under the same conditions, the competitive inhibition of labeling using excess Sal-AMS is not observed, suggesting that the modification at the 2′-OH group of the adenosine in AA-AMS might affect the cell permeability of the compounds. Given the high hydrophilicity of the bisubstrate AMS scaffold, it is reasonable to conclude that the BPyne component increases hydrophobicity and facilitates accumulation in live *E. coli*. In the present study, we investigated the influence of the introduction of several functional groups at the 2′-OH group of the AMS scaffold on both the binding affinities for A-domains and cell permeability (Figure 2c and Figure 3). We selected the ʟ-Phe-selective A-domain of the gramicidin S synthetase GrsA, which was previously demonstrated to be selectively labeled with ʟ-Phe-AMS-BPyne (**3**) in *Aneurinibacillus migulanus* ATCC 9999 [19]. Intracellular competitive ABPP of GrsA using ʟ-Phe-AMS-BPyne was also performed to reveal the cell permeability of 2′-OH-modified ʟ-Phe-AMS derivatives.

## Results and Discussion

**Scheme 1:**
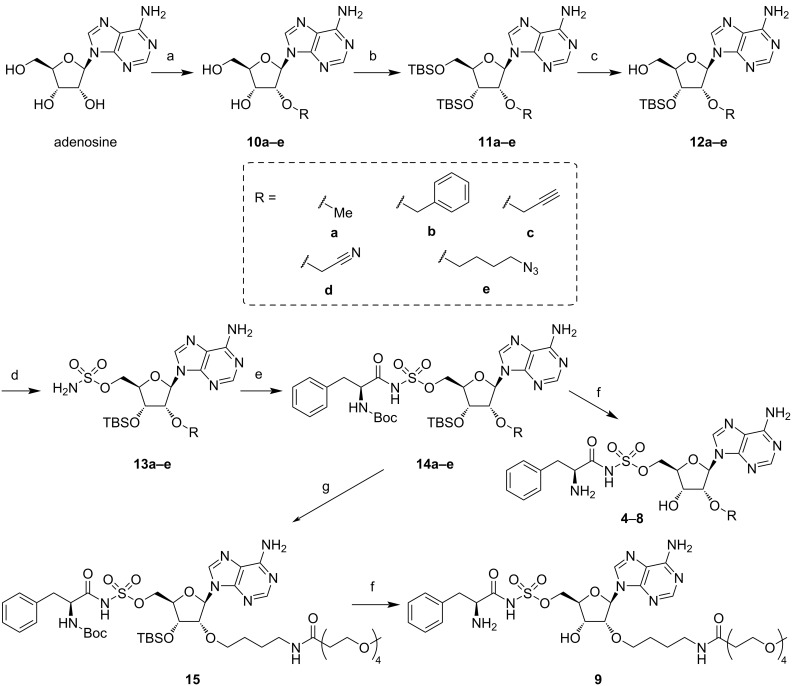
Synthesis of 2′-OH-substituted ʟ-Phe-AMS derivatives. Reagents and conditions: (a) NaH, TBAI, R–X (a: MeI, b: BnBr, c: BrCH_2_CCH_3_, d: BrCH_2_CN, e: Br(CH_2_)_4_N_3_), DMF, rt, 45% (**10a**), 59% (**10b**), 45% (**10c**), 21% (**10d**), and 45% (**10e**); (b) TBSCl, imidazole, DMAP, DMF, rt, 83% (**11a**), 78% (**11b**), 57% (**11c**), 62% (**11d**), and 65% (**11e**); (c) 25% TFA in THF aq, 0 °C, 77% (**12a**), 77% (**12b**), 50% (**12c**), 96% (**12d**), and 75% (**12e**); (d) NaH, NH_2_SO_2_Cl, DME, 0 °C to rt, 86% (**13a**), 81% (**13b**), 92% (**13c**), 78% (**13d**), and 62% (**13e**); (e) Boc-ʟ-Phe-OSu, Cs_2_CO_3_, DMF, rt.; (f) 80% aqueous TFA, rt, two steps 95% (**4**), 94% (**5**), 56% (**6**), 88% (**7**), 58% (**8**), 69% (**9**); (g) methyl-Peg_4_-NHS ester, Cs_2_CO_3_, DMF, rt.

To investigate the influence of the introduction of different alkyl groups at the 2′-OH, we prepared ʟ-Phe-AMS derivatives **4**–**9**. As the compounds **6**, **8**, and **9** were synthesized previously [20], we designed and synthesized three new ʟ-Phe-AMS derivatives containing methyl (**4**), benzyl (**5**), and cyanomethyl (**7**) groups at the 2′-OH. The synthetic routes to compounds **4**–**9** are shown in Scheme 1. The 2′-OH of adenosine was alkylated with several alkyl halides in the presence of sodium hydride (NaH). Both the 3′-OH and 5′-OH groups of compounds **10a**–**e** were protected by a TBS group, followed by the selective deprotection of the 5′-OH group using 25% trifluoroacetic acid in tetrahydrofuran. Subsequently, the 5′-OH group of compounds **12a**–**e** were reacted with sulfamoyl chloride in the presence of NaH. Compounds **13a**–**e** were coupled to pre-activated Boc-ʟ-Phe-OSu in the presence of Cs_2_CO_3_. Removal of the Boc and TBS groups of compounds **14a**–**e** yielded the desired ʟ-Phe-AMS derivatives **4**, **5**, and **7**.

We first determined the binding affinities of ʟ-Phe-AMS derivatives **4**–**9** for the A-domain of GrsA using a previously developed competitive enzyme-linked immunosorbent assay technique for A-domains in NRPSs [14], which allowed us to measure the dissociation constant (*K*_d_) of the test compounds (Figure S1a, Supporting Information File 1). The ʟ-Phe-AMS-biotin probe was immobilized on a streptavidin-coated 96-well plate and incubated with recombinant His_6_-tagged GrsA in the presence or absence of inhibitors (Figure S1b, Supporting Information File 1). After washing, the wells were incubated with an anti-His_6_ tag antibody and subsequently with a horseradish peroxidase-conjugated secondary antibody. The amount of GrsA bound to ʟ-Phe-AMS-biotin was determined by measuring the absorbance of *o*-phenylenediamine dihydrochloride at 492 nm. The *K*_d_ values of the ʟ-Phe-AMS derivatives are listed in Table 1. Compared with the binding affinity of ʟ-Phe-AMS **1** (*K*_d_ value, 11.4 ± 3.4 nM) for the A-domain of GrsA, all tested compounds showed slightly decreased binding affinities. Among them, inhibitors **4** (23.9 ± 0.7 nM), **7** (16.6 ± 0.6 nM), and **8** (30.2 ± 4.2 nM) displayed *K*_d_ values comparable to that of ʟ-Phe-AMS **1**, suggesting that the substitution of small functional groups such as methyl and cyanomethyl groups has a minor effect on the *K*_d_ values (Table 1 and Figures S2 and S3, Supporting Information File 1). In contrast, inhibitors **6** and **9** exhibited lower affinity than inhibitors **1**, **2**, **4**, **5**, and **7**. This result suggests that the incorporation of the pegylated functionality at the 2′-OH group of the adenosine skeleton particularly perturbs binding via steric hindrance.

**Table 1:** *K*_d_ values for the A-domain of gramicidin S synthetase^a^.

Compounds	*K*_d_ [nM]

ʟ-Phe-AMS^b^ (**1**)	11.4 ± 3.4
ᴅ-Phe-AMS^b^ (**2**)	26.4 ± 4.1
ʟ-Phe-AMS-Me (**4**)	23.9 ± 0.7
ʟ-Phe-AMS-Bn (**5**)	33.6 ± 0.9
ʟ-Phe-AMS-alkyne^b^ (**6**)	60.0 ± 3.6
ʟ-Phe-AMS-CN (**7**)	16.6 ± 0.6
ʟ-Phe-AMS-azide^b^ (**8**)	30.2 ± 4.2
ʟ-Phe-AMS-peg^b^ (**9**)	85.5 ± 10.7

^a^*K*_d_ values were determined via competitive enzyme-linked immunosorbent assays using ʟ-Phe-AMS-biotin at room temperature in 20 mM Tris (pH 8.0), 1 mM MgCl_2_, 1 mM TCEP, and 0.0025% NP-40. ^b^*K*_d_ values were obtained from the study of Ishikawa et al. [20]. The assay conditions were identical to those of the present study.

**Figure 4:**
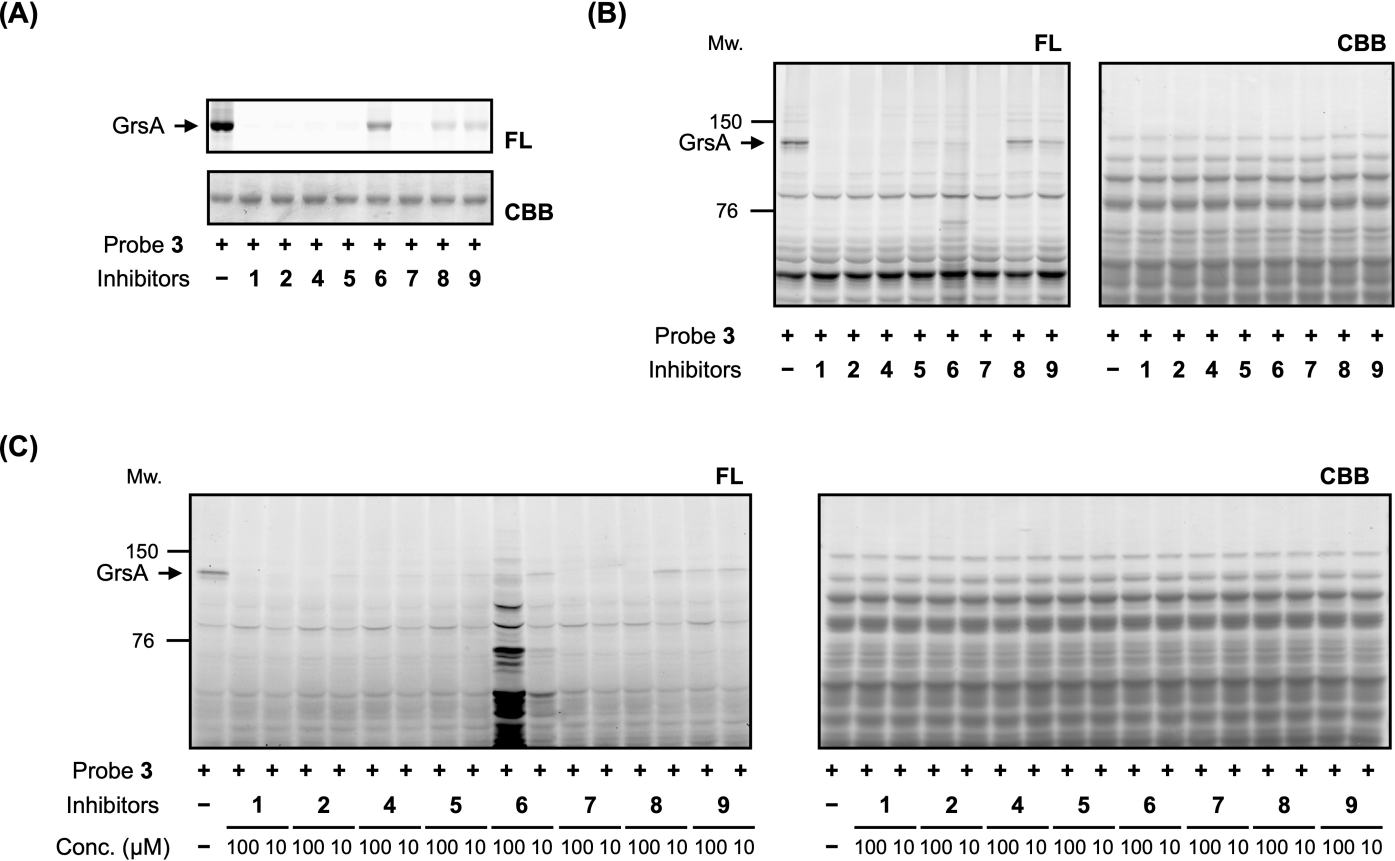
Competitive labeling experiments of GrsA using probe **3** in the presence of ʟ-Phe-AMS inhibitors. (A) Labeling of recombinant GrsA with probe **3** in either the absence or presence of ʟ-Phe-AMS inhibitors. Recombinant GrsA (1 µM) was pre-incubated with or without ʟ-Phe-AMS inhibitors **1**, **2**, or **4**–**9** (100 µM) and then treated with probe **3** (1 µM). (B) Competitive labeling assay of endogenous GrsA with probe **3** using the lysates of *Aneurinibacillus migulanus* ATCC 9999. The lysates were pre-incubated with or without ʟ-Phe-AMS inhibitors **1**, **2,** or **4**–**9** (100 µM) and then treated with probe **3** (1 µM). (C) Intracellular competitive labeling of GrsA in *A. migulanus* ATCC 9999. *A. migulanus* ATCC 9999 in YPG medium was treated with probe **3** (10 µM) in either the absence or presence of ʟ-Phe-AMS inhibitors **1**, **2**, or **4**–**9** (10 or 100 µM). GrsA, gramicidin S synthetase; AMS, 5′-*O*-sulfamoyladenosine; FL, fluorescent gel; CBB; Coomassie brilliant blue.

Next, we investigated whether ʟ-Phe-AMS derivatives could competitively inhibit the labeling of recombinant GrsA using the ABP ʟ-Phe-AMS-BPyne (**3**). GrsA (1 µM) was incubated with probe **3** (1 µM) in the absence or presence of either inhibitor **1**, **2**, or **4**–**9** (100 µM). The mixtures were exposed to ultraviolet light at 365 nm to form a covalent bond between GrsA and the benzophenone moiety in probe **3**. The samples were then reacted with TAMRA-N_3_ (structure shown in Figure S4, Supporting Information File 1) under copper(I)-catalyzed azide–alkyne cycloaddition conditions [21] and subjected to sodium dodecyl sulfate-polyacrylamide gel electrophoresis coupled with in-gel fluorescence scanning (excitation wavelength, 532 nm; emission wavelength, 580 nm). The labeling of GrsA by probe **3** was completely suppressed by the addition of inhibitors **1**, **2**, **4**, **5**, and **7** (Figure 4a). In contrast, inhibitors **6**, **8**, and **9** moderately inhibited labeling, which was consistent with the decreased *K*_d_ values of these compounds. Subsequently, we conducted competitive labeling experiments for endogenous GrsA in the proteome of the gramicidin S-producer *A. miglanus* ATCC 9999. The cellular lysates of strain ATCC 9999 were treated with probe **3** (1 µM) in the absence or presence of inhibitor **1**, **2**, or **4**–**9** (100 µM), irradiated at 365 nm, and subjected to the click reaction with TAMRA-N_3_. In-gel fluorescence scanning revealed that inhibitors **1**, **2**, **4**, and **7** completely suppressed GrsA labeling by probe **3** in the proteomic environment (Figure 4b). Unlike the results obtained for the labeling of purified recombinant GrsA, inhibitor **5** moderately inhibited labeling at 100 µM.

Finally, we investigated whether the inhibitors could penetrate cells and inhibit intracellular GrsA labeling in *A. miglanus* ATCC 9999. In this study, *A. migulanus* ATCC 9999 cells were grown at 37 °C in YPG medium for 24 h, harvested, and washed with phosphate-buffered saline. The bacterial suspension was then treated with probe **3** (10 µM) in either the absence or presence of inhibitors **1**, **2**, or **4**–**9** (10 or 100 µM). Inhibitors **1**, **2**, **4**, **5**, **7**, and **8** completely inhibited the labeling of endogenous GrsA at high concentrations (100 µM), suggesting that these inhibitors can penetrate cells (Figure 4c). Notably, inhibitors **1** and **7** efficiently inhibited the labeling of GrsA at 10 µM. The incorporation of the nitrile group at the 2’-OH group of the adenosine skeleton is expected to provide chemical properties that would allow the compound to retain its binding affinity and cell permeability. Overall, these results indicate that a 2′-OH modification with a cyanomethyl group represents a useful AMS scaffold for intracellular NRPS inhibition.

## Conclusion

In this study, we investigated the effect of a 2′-OH modification in an AMS scaffold on the binding affinities for A-domains and cell permeability. Our experiments demonstrated that inhibitor **7**, harboring a cyanomethyl group at the 2′-OH, showed a *K*_d_ value comparable to that of the original ʟ-Phe-AMS **1**. In addition, intracellular competitive ABPP suggested that the inhibitor **7** can penetrate cells. The application of this new scaffold to NRPS inhibitors involved in the production of virulence factors could thus facilitate the development of new antibiotics.

## Supporting Information

File 1 Additional Figures, experimental part and NMR spectra.

## Data Availability

All data that supports the findings of this study is available in the published article and/or the supporting information to this article.
