# Supplementary material for: Development of a chemical scaffold for inhibiting nonribosomal peptide synthetases in live bacterial cells
Source: Beilstein J Org Chem. 2024 Feb 26;20:445–51. doi: 10.3762/bjoc.20.39 (PMC10910458; doi:10.3762/bjoc.20.39)

BEILSTEIN JOURNAL OF ORGANIC CHEMISTRY

Supporting Information

for

Development of a chemical scaffold for inhibiting nonribosomal peptide synthetases in live bacterial cells

Fumihiro Ishikawa, Sho Konno, Hideaki Kakeya and Genzoh Tanabe

Beilstein J. Org. Chem. **2024,** *20,* 445–451. doi:10.3762/bjoc.20.39

Additional Figures, experimental part and NMR spectra

License and Terms: This is a supporting information file under the terms of the Creative Commons Attribution License (https://creativecommons.org/licenses/by/4.0). Please note that the reuse, redistribution and reproduction in particular requires that the author(s) and source are credited and that individual graphics may be subject to special legal provisions.
The license is subject to the *Beilstein Journal of Organic Chemistry* terms and conditions: (https://www.beilstein-journals.org/bjoc/terms)

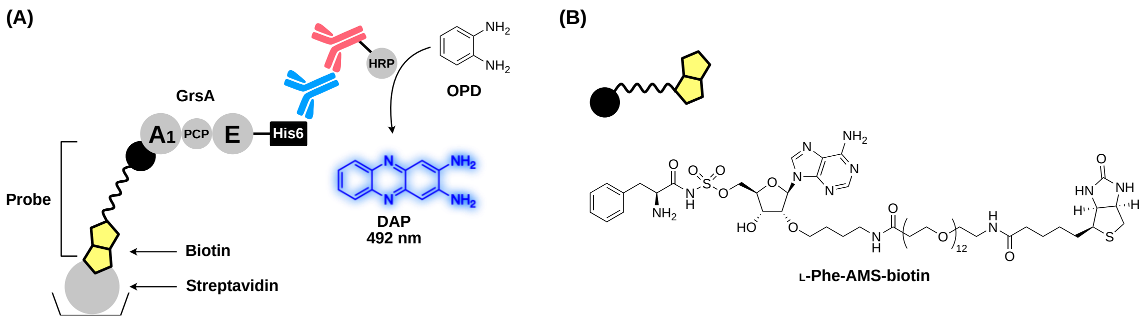

Figure S1. (A) Enzyme-linked immunosorbent assay (ELISA) for the binding of adenylation domains of nonribosomal peptide synthetases.[1,2,3] (B) A structure of the chemical probe described in this ELISA protocol.[1,2,3]

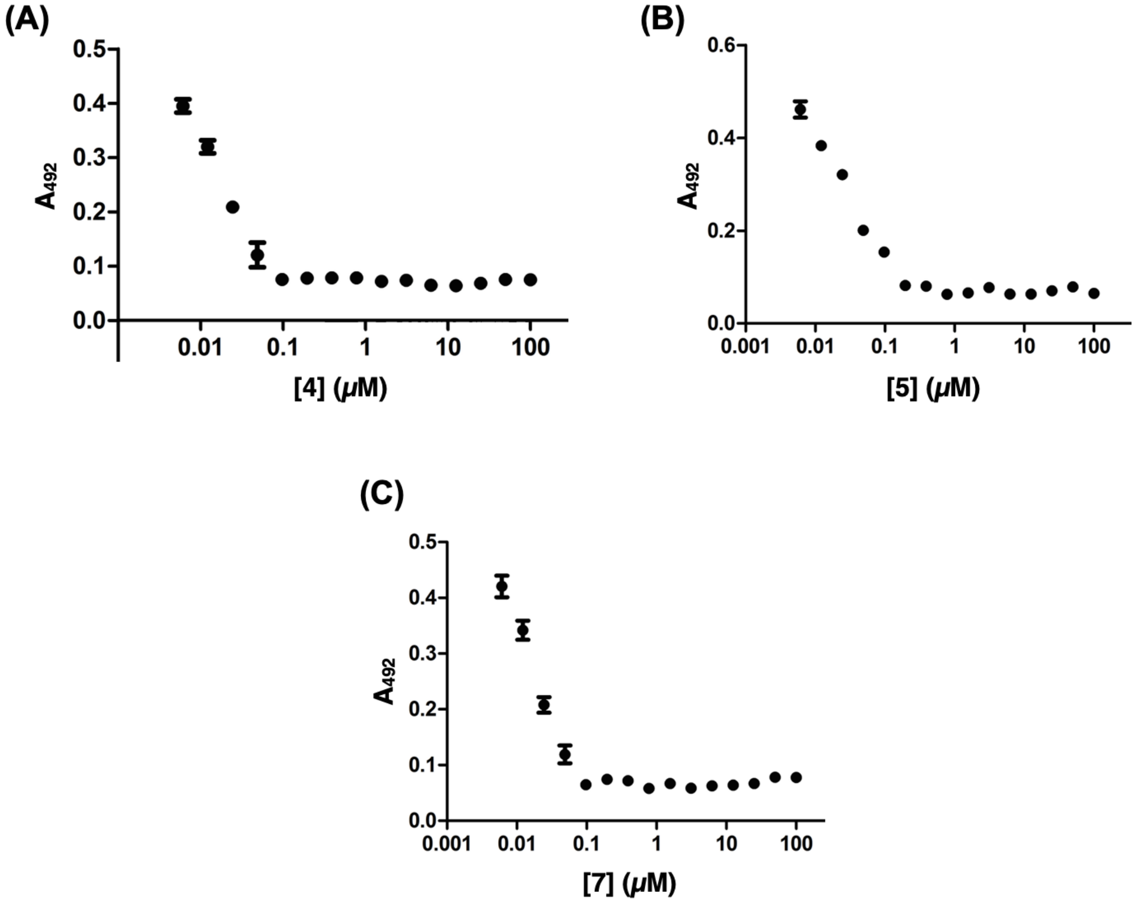

Figure S2. Binding of GrsA with (A) inhibitor 4, (B) inhibitor 5, and (C) inhibitor 7 by competitive ELISA with L-Phe-AMS-biotin. Streptavidin-coated wells treated with L-Phe-AMS-biotin (1.5 μg/mL) were incubated with GrsA (0.875 μg/mL, 6.89 nM) in either the absence or presence of inhibitor **4** (6.1 nM−100 μM), **5** (6.1 nM−100 μM), or **7** (6.1 nM−100 μM).

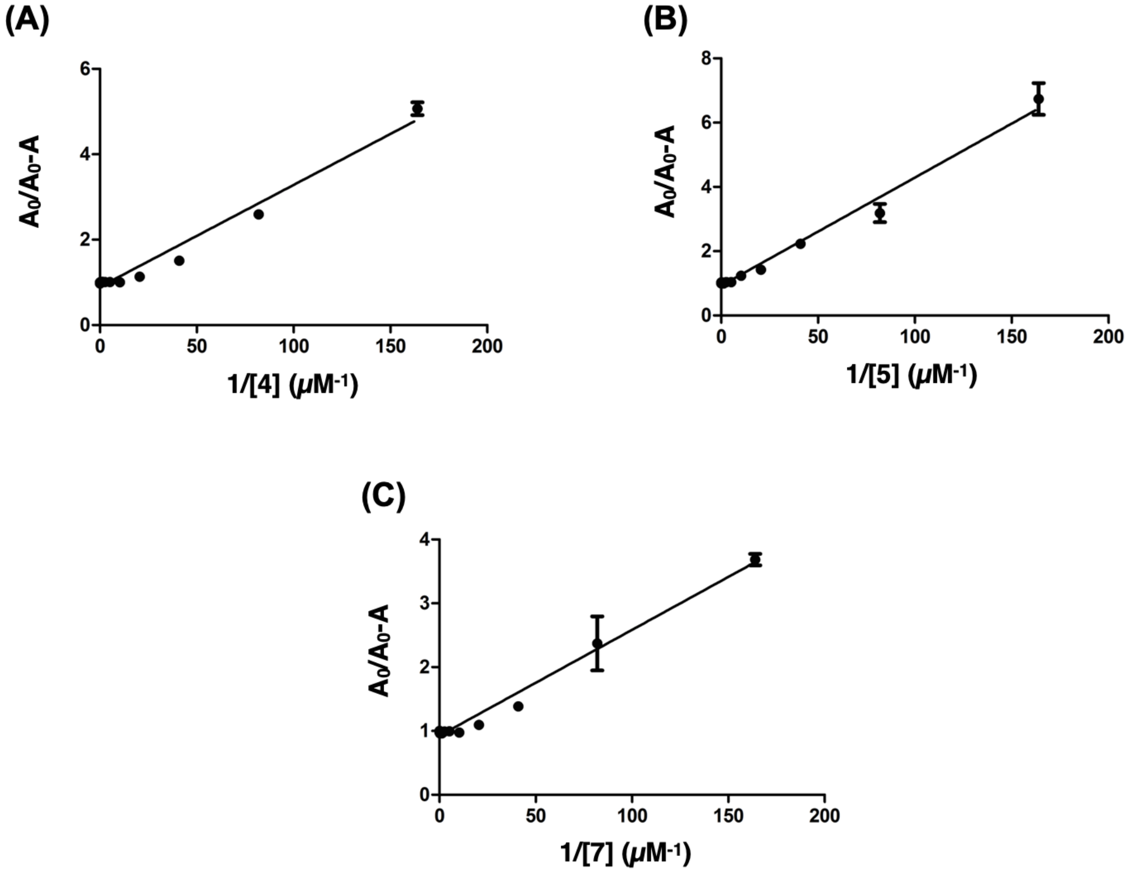

Figure S3. Klotz plots to determine K_d values of GrsA with (A) inhibitor 4, (B) inhibitor 5, and (C) inhibitor 7. The results were analyzed by a Klotz plot (a double reciprocal plot of the inhibition concentration vs the ratio of inhibitor-bound enzyme to the total added enzyme) and the slope of the line yielding the K_d for the probe. All assays were performed in duplicates.

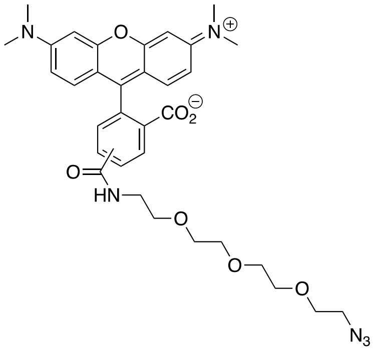

Figure S4. Structure of TAMRA-N_3.

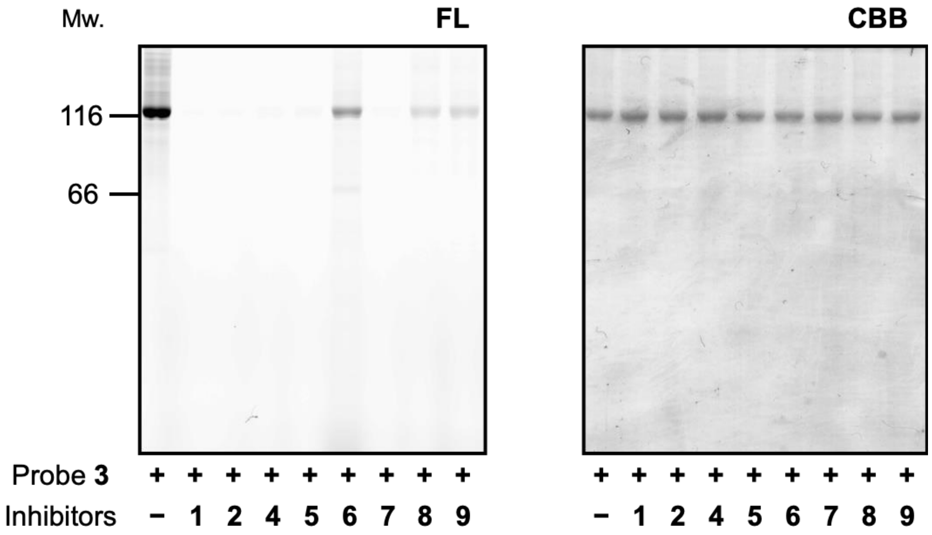

Figure S5. Full gel image of Figure 4A.

Chemical synthetic procedures

General synthetic methods: All commercial reagents were used as provided unless otherwise indicated. Compounds **1**[4], **2**[2], **3**[5], **6**[2], **8**[2], **9**[2], **10a–12a**[6], and **10b**[7] were known compounds. These compounds were prepared according to published literature procedures. All reactions were carried out under an atmosphere of nitrogen in dry solvents with oven-dried glassware and constant magnetic stirring unless otherwise noted. ^{1}H NMR spectra were recorded at 500 MHz. ^{13}C NMR spectra were recorded at 125 MHz on JEOL NMR spectrometers and standardized to the NMR solvent signal as reported by Gottlieb.[8] Multiplicities are given as s = singlet, d = doublet, t = triplet, q = quartet, dd = doublet of doublets, ddd = doublet of triplets, br = broad signal, m = multiplet using integration and coupling constant in Hertz. TLC analysis was performed using Silica Gel 60 F254 plates (Merck) and visualization was accomplished with ultraviolet light (λ = 254 nm) and/or the appropriate stain [phosphomolybdic acid, iodine, ninhydrin, and potassium permanganate]. Silica gel chromatography was carried out with SiliaFlash F60 230-400 mesh (Silicycle), according to the method of Still.[9] Mass spectral data were obtained using a LCMS-IT-TOF mass spectrometer (Shimadzu).

2-(((2*R*,3*R*,4*R*,5*R*)-2-(6-Amino-9*H*-purin-9-yl)-4-hydroxy-5-(hydroxymethyl) tetrahydrofuran-3-yl)oxy)acetonitrile (10d)

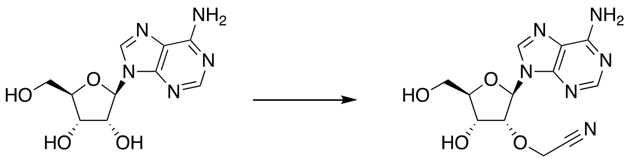

10d

NaH (180 mg of a 60% suspension in mineral oil, 4.49 mmol) was added to the solution of adenosine (1.00 g, 3.75 mmol) in DMF (30 mL). The solution was stirred at 0 °C for 30 min and bromoacetonitrile (275 µL, 4.13 mmol) and TBAI (277 mg, 0.75 mmol) were added. The reaction mixture was stirred at room temperature for 9 h. The mixture was evaporated to dryness. The residue was purified by flash chromatography (20:1 to 10:1 $CHCl_3$/MeOH) to afford compound **10d** as a white solid (242 mg, 21%). ^{1}H NMR (500 MHz, Methanol-*d*$_4$) δ 8.64 (s, 1H), 8.42 (s, 1H), 6.22 (d, *J* = 4.4 Hz, 1H), 4.67 – 4.51 (m, 4H), 4.20 – 4.13 (m, 1H), 3.91 (dd, *J* = 12.4, 2.8 Hz, 1H), 3.80 (dd, *J* = 12.4, 3.2 Hz, 1H). ^{13}C NMR (125 MHz, Methanol-*d*$_4$) δ 152.1, 149.5, 145.7, 143.6, 120.4, 117.3, 88.5, 87.4, 84.3, 70.3, 62.0, 57.0. HRMS (ESI−): [M−H]$^{-}$ calcd for $C_{12}H_{13}N_6O_4$, 305.0998; found, 305.0991.

9-((2*R*,3*R*,4*R*,5*R*)-3-(Benzyloxy)-4-((*tert*-butyldimethylsilyl)oxy)-5-(((*tert*-butyldimethylsilyl)oxy)methyl)tetrahydrofuran-2-yl)-9*H*-purin-6-amine (11b)

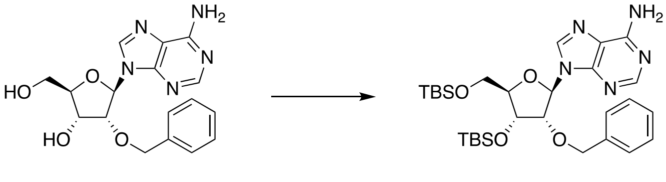

10b **11b**

To a solution of compound **10b** (200 mg, 0.56 mmol), imidazole (228 mg, 3.36 mmol) and DMAP (10.0 mg, 0.08 mmol) in DMF (6 mL) was added TBSCl (211 mg, 1.40 mmol) at 0 °C. The solution was stirred at room temperature for 16 h. The reaction mixture was diluted with EtOAc. The mixture was washed with 5% citric acid, 5% $NaHCO_3$, and brine. The organic layer was dried over Na_2SO_4 and evaporated to dryness. The residue was purified by flash chromatography (50:1 $CHCl_3$/MeOH) to afford compound **11b** as a white solid (255 mg, 78%). ^{1}H NMR (500 MHz, $CDCl_3$) δ 8.39 (s, 1H), 8.09 (s, 1H), 6.42 (s, 2H), 6.25 (d, *J* = 4.3 Hz, 1H), 4.76 (d, *J* = 12.1 Hz, 1H), 4.67 (d, *J* = 12.1 Hz, 1H), 4.57 (t, *J* = 4.8 Hz, 1H), 4.45 (t, *J* = 4.5 Hz, 1H), 4.24 – 4.17 (m, 1H), 4.04 (dd, *J* = 11.4, 3.8 Hz, 1H), 3.82 (dd, *J* = 11.5, 2.8 Hz, 1H), 0.97 (s, 9H), 0.96 (s, 9H), 0.15 – 0.12 (m, 12H). ^{13}C NMR (125 MHz, $CDCl_3$) δ 155.7, 152.9, 149.6, 139.2, 137.2, 128.3, 127.9, 127.8, 120.0, 86.8, 85.2, 80.7, 77.3, 77.0, 76.8, 72.2, 70.0, 62.0, 26.0, 25.7, 18.5, 18.1, -4.6, –4.9, –5.4, –5.5. HRMS (ESI–): $[M-H]^-$ calcd for $C_{29}H_{46}N_5O_4Si_2$, 584.3088; found, 584. 3097.

2-(((2*R*,3*R*,4*R*,5*R*)-2-(6-Amino-9*H*-purin-9-yl)-4-((*tert*-butyldimethylsilyl)oxy)-5-(((*tert*-butyldimethylsilyl)oxy)methyl)tetrahydrofuran-3-yl)oxy)acetonitrile (11d)

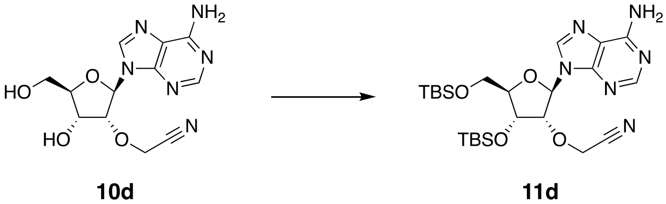

10d **11d**

To a solution of compound **10d** (117 mg, 0.38 mmol), imidazole (155 mg, 2.28 mmol) and DMAP (7.3 mg, 0.06 mmol) in DMF (4 mL) was added TBSCl (143 mg, 0.95 mmol) at 0 °C. The solution was stirred at room temperature for 11 h. The reaction mixture was diluted with EtOAc. The mixture was washed with 5% citric acid, 5% $NaHCO_3$, and brine. The organic layer was dried over Na_2SO_4 and evaporated to dryness. The residue was purified by flash chromatography (100:1 $CHCl_3$/MeOH) to afford compound **11d** as a white solid (127 mg, 62%). ^{1}H NMR (500 MHz, $CDCl_3$) δ 8.31 (s, 1H), 8.15 (s, 1H), 6.50 (s, 2H), 6.11 (d, *J* = 3.3 Hz, 1H), 4.64 – 4.55 (m, 1H),

4.57 – 4.49 (m, 3H), 4.13 – 4.05 (m, 1H), 3.99 (dd, *J* = 11.7, 3.1 Hz, 1H), 3.76 (dd, *J* = 11.6, 2.6 Hz, 1H), 0.91 (s, 9H), 0.90 (s, 9H), 0.13 (s, 3H), 0.11 (s, 3H), 0.084 (s, 3H), 0.078 (s, 3H). ^{13}C NMR (125 MHz, $CDCl_3$) δ 155.8, 153.1, 149.2, 138.8, 120.1, 115.5, 86.7, 84.5, 82.3, 69.6, 61.3, 55.7, 25.9, 25.6, 18.4, 18.0, –4.6, –4.9, –5.5. HRMS (ESI−): $[M−H]^-$ calcd for $C_{24}H_{41}N_6O_4Si_2$, 533.2728; found, 533.2755.

((2*R*,3*R*,4*R*,5*R*)-5-(6-Amino-9*H*-purin-9-yl)-4-(benzyloxy)-3-((*tert*-butyldimethylsilyl)oxy)tetrahydrofuran-2-yl)methanol (12b)

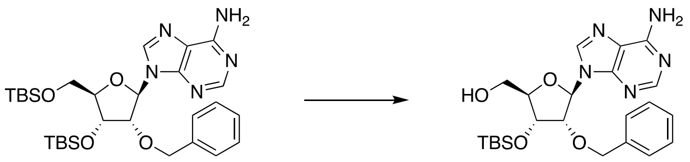

11b **12b**

Compound **11b** (200 mg, 0.34 mmol) was dissolved in a 1:1 (v/v) mixture of a 50% aqueous solution of TFA (2mL) and THF (2 mL) at 0 °C. After 6 h, the flask was placed on the rotary evaporator and the TFA, THF and H_2O were removed at reduced pressure. The residue was purified by flash chromatography (100:1 to 50:1 $CHCl_3$/MeOH) to afford compound **12b** as a white solid (123 mg, 77%). ^{1}H NMR (500 MHz, $CDCl_3$) δ 8.12 (s, 1H), 7.75 (s, 1H), 7.03 – 6.95 (m, 3H), 6.93 (dt, *J* = 6.4, 1.8 Hz, 2H), 6.72 (d, *J* = 11.7 Hz, 1H), 6.47 (s, 2H), 5.83 (d, *J* = 7.9 Hz, 1H), 4.75 (dd, *J* = 8.0, 4.5 Hz, 1H), 4.61 – 4.55 (m, 2H), 4.22 – 4.12 (m, 2H), 3.89 (dd, *J* = 13.1, 1.6 Hz, 1H), 3.76 – 3.65 (m, 1H), 0.94 (s, 9H), 0.15 (s, 3H), 0.14 (s, 3H). ^{13}C NMR (125 MHz, $CDCl_3$) δ 156.0, 151.8, 148.1, 140.6, 136.7, 127.8, 127.7, 127.6, 121.1, 89.9, 89.2, 78.7, 72.4, 71.7, 63.0, 25.8, 18.2, –4.6, –4.9. HRMS (ESI−): $[M−H]^-$ calcd for $C_{23}H_{41}N_5O_4Si$, 470.2224; found, 470.2223.

2-(((2*R*,3*R*,4*R*,5*R*)-2-(6-Amino-9*H*-purin-9-yl)-4-((*tert*-butyldimethylsilyl)oxy)-5-(hydroxymethyl)tetrahydrofuran-3-yl)oxy)acetonitrile (12d)

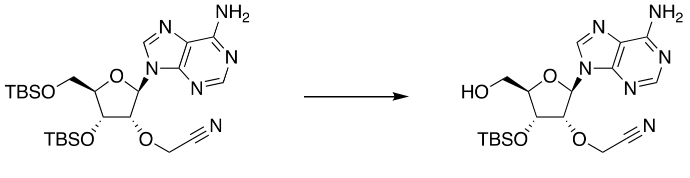

11d **12d**

Compound **11d** (100 mg, 0.19 mmol) was dissolved in a 1:1 (v/v) mixture of a 50% aqueous solution of TFA (2mL) and THF (2 mL) at 0 °C. After 6 h, the flask was placed on the rotary

evaporator and the TFA, THF and H_2O were removed at reduced pressure. The residue was purified by flash chromatography (100:1 to 50:1 $CHCl_3$/MeOH) to afford compound **12d** as a white solid (77 mg, 96%). ^1H NMR (500 MHz, $CDCl_3$) δ 8.27 (s, 1H), 7.88 (s, 1H), 6.54 (s, 2H), 5.92 (d, *J* = 7.4 Hz, 1H), 4.90 (dd, *J* = 7.3, 4.6 Hz, 1H), 4.61 (dd, *J* = 4.6, 1.1 Hz, 1H), 4.24 – 4.18 (m, 2H), 4.13 (d, *J* = 15.8 Hz, 1H), 3.93 (dd, *J* = 13.2, 1.7 Hz, 1H), 3.71 (d, *J* = 13.1 Hz, 1H), 0.93 (s, 9H), 0.13 (s, 3H), 0.12 (s, 3H). ^{13}C NMR (125 MHz, $CDCl_3$) δ 156.3, 152.5, 148.5, 140.6, 121.1, 115.1, 89.3, 88.8, 81.5, 71.4, 62.6, 55.9, 25.7, 18.1, –4.6, –4.8. HRMS (ESI–): $[M-H]^-$ calcd for $C_{18}H_{27}N_6O_4S$, 419.1863; found, 419.1865.

((2*R*,3*R*,4*R*,5*R*)-5-(6-Amino-9*H*-purin-9-yl)-3-((*tert*-butyldimethylsilyl)oxy)-4-methoxytetrahydrofuran-2-yl)methyl sulfamate (13a)

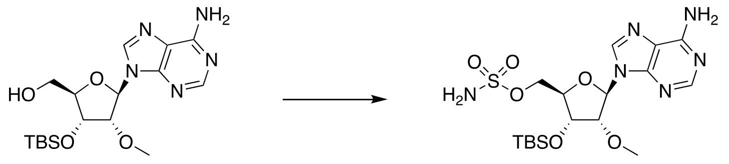

12a **13a**

NaH (18.0 mg of a 60 % suspension in mineral oil, 0.45 mmol) was added to a solution of compound **12a** (120 mg, 0.30 mmol) in DME (3 mL). The solution was stirred at 0 °C for 30 min and sulfamoyl chloride (52 mg, 0.45 mmol) was added. The reaction mixture was stirred at room temperature for 3 h. The reaction was quenched with MeOH (10 mL) and the mixture was evaporated to dryness. The residue was purified by flash chromatography (40:1 to 10:1 $CHCl_3$/MeOH) to afford compound **13a** as a white solid (122 mg, 86%). ^1H NMR (500 MHz, Methanol-d_4) δ 8.30 (s, 1H), 8.21 (s, 1H), 6.14 (d, *J* = 5.0 Hz, 1H), 4.66 (t, *J* = 4.5 Hz, 1H), 4.48 – 4.36 (m, 2H), 4.36 – 4.24 (m, 2H), 3.42 (s, 3H), 0.94 (s, 9H), 0.16 (s, 3H), 0.15 (s, 3H). ^{13}C NMR (125 MHz, Methanol-d_4) δ 157.2, 154.0, 150.5, 140.9, 120.4, 87.9, 84.1, 84.1, 79.4, 71.9, 69.0, 58.9, 26.2, 18.9, –4.6, –4.7. HRMS (ESI–): $[M-H]^-$ calcd for $C_{17}H_{29}N_6O_6SSi$, 473.1639; found, 473.1622.

((2*R*,3*R*,4*R*,5*R*)-5-(6-Amino-9*H*-purin-9-yl)-4-(benzyloxy)-3-((*tert*-butyldimethylsilyl) oxy)tetrahydrofuran-2-yl)methyl sulfamate (13b)

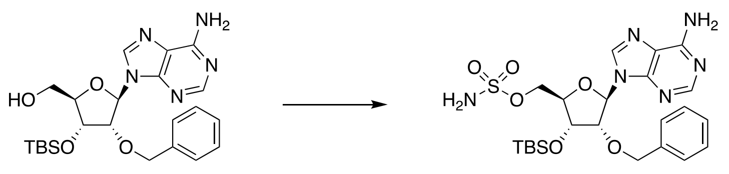

12b **13b**

NaH (15.0 mg of a 60 % suspension in mineral oil, 0.38 mmol) was added to a solution of compound **12b** (120 mg, 0.25 mmol) in DME (3 mL). The solution was stirred at 0 °C for 30 min and sulfamoyl chloride (44 mg, 0.38 mmol) was added. The reaction mixture was stirred at room temperature for 3 h. The reaction was quenched with MeOH (10 mL) and the mixture was evaporated to dryness. The residue was purified by flash chromatography (50:1 to 20:1 $CHCl_3$/MeOH) to afford compound **13b** as a white solid (112 mg, 81%). ^{1}H NMR (500 MHz, Methanol-d_4) δ 8.15 (s, 1H), 8.11 (s, 1H), 7.11 (s, 5H), 6.17 – 6.10 (m, 1H), 4.70 – 4.61 (m, 3H), 4.50 – 4.37 (m, 2H), 4.32 (dt, J = 5.0, 3.7 Hz, 2H), 0.96 (s, 9H), 0.18 (s, 3H), 0.15 (s, 3H). ^{13}C NMR (125 MHz, Methanol-d_4) δ 157.2, 153.7, 150.4, 141.0, 138.6, 129.1, 129.0, 128.8, 120.4, 88.0, 84.8, 81.5, 73.7, 72.4, 69.2, 26.3, 19.0, –4.4, –4.8. HRMS (ESI–): [M–H]$^{-}$ calcd for $C_{23}H_{33}N_6O_6SSi$, 549.1952; found, 549.1960.

((2*R*,3*R*,4*R*,5*R*)-5-(6-Amino-9*H*-purin-9-yl)-3-((*tert*-butyldimethylsilyl)oxy)-4-(cyanomethoxy)tetrahydrofuran-2-yl)methyl sulfamate (13d)

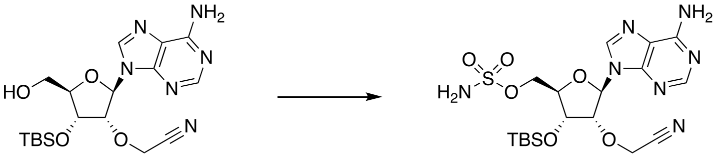

12d **13d**

NaH (11.0 mg of a 60 % suspension in mineral oil, 0.27 mmol) was added to a solution of compound **12d** (75 mg, 0.18 mmol) in DME (2 mL). The solution was stirred at 0 °C for 30 min and sulfamoyl chloride (31 mg, 0.27 mmol) was added. The reaction mixture was stirred at room temperature for 3 h. The reaction was quenched with MeOH (5 mL) and the mixture was evaporated to dryness. The residue was purified by flash chromatography (30:1 to 10:1 $CHCl_3$/MeOH) to afford compound **13d** as a white solid (69 mg, 78%). ^{1}H NMR (500 MHz, Methanol-d_4) δ 8.30 (s, 1H), 8.22 (s, 1H), 6.19 (d, J = 4.5 Hz, 1H), 4.79 (t, J = 4.7 Hz, 1H), 4.75 (t, J = 4.6 Hz, 1H), 4.55 (d, J = 1.9 Hz, 2H), 4.45 (dd, J = 12.2, 5.0 Hz, 1H), 4.34 – 4.28 (m, 2H), 0.97 (s, 9H), 0.20 (s, 6H). ^{13}C NMR (125 MHz, Methanol-d_4) δ 157.2, 153.9, 150.3, 140.9, 120.5, 117.0, 87.9, 83.8, 82.8, 71.7, 68.6, 56.8, 26.1, 18.8, –4.7, –4.9. HRMS (ESI–): [M–H]$^{-}$ calcd for $C_{18}H_{28}N_7O_6SSi$, 498.1591; found, 498.1582.

((2*R*,3*R*,4*R*,5*R*)-5-(6-Amino-9*H*-purin-9-yl)-3-hydroxy-4-methoxytetrahydrofuran-2-yl)methyl (*L*-phenylalanyl)sulfamate (4)

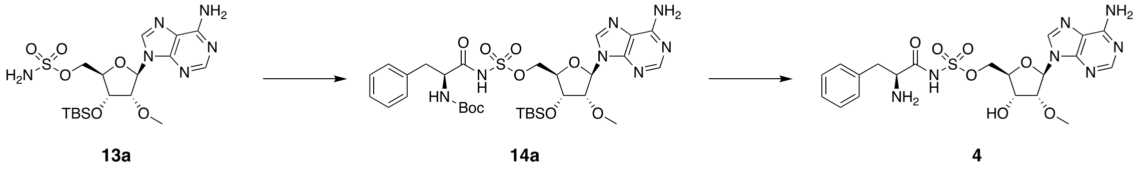

Boc-Phe-OSu (33 mg, 0.09 mmol) and Cs_2CO_3 (59 mg, 0.18 mmol) were added to a solution of compound **13a** (30 mg, 0.06 mmol) in DMF (1.0 mL). The solution was stirred at room temperature for 2 h. The reaction mixture was diluted with EtOAc. The mixture was washed with 5% citric acid, 5% $NaHCO_3$, and brine. The organic layer was dried over Na_2SO_4 and evaporated to dryness. The residue was used to next step without further purifications. Crude compound **14a** was dissolved in a 4:1 (v/v) mixture of TFA and H_2O at room temperature. After 6 h, the flask was placed on the rotary evaporator and the TFA and H_2O were removed at reduced pressure. The residue was purified by flash chromatography (10:1 to 5:1 $CHCl_3$/MeOH) to afford compound **4** as a white solid (29 mg, 95%). ^1H NMR (500 MHz, Methanol-d_4) δ 8.49 (s, 1H), 8.20 (s, 1H), 7.29 – 7.23 (m, 4H), 7.20 – 7.14 (m, 1H), 6.17 (d, J = 4.7 Hz, 1H), 4.48 (t, J = 4.8 Hz, 1H), 4.37 (dd, J = 11.3, 2.9 Hz, 1H), 4.34 – 4.24 (m, 3H), 3.91 (dd, J = 8.2, 4.9 Hz, 1H), 3.50 (s, 3H), 3.04 (dd, J = 14.4, 8.2 Hz, 1H), 2.90 (dd, J = 14.0, 8.5 Hz, 1H). ^{13}C NMR (125 MHz, Methanol-d_4) δ 175.6, 157.2, 154.0, 150.6, 141.1, 136.6, 130.6, 130.6, 129.9, 129.9, 128.4, 128.3, 120.1, 87.6, 85.0, 84.3, 70.6, 68.9, 59.0, 58.3, 38.6. HRMS (ESI−): $[M-H]^-$ calcd for $C_{20}H_{24}N_7O_7S$, 506.1458; found, 506.1441.

((2*R*,3*R*,4*R*,5*R*)-5-(6-Amino-9*H*-purin-9-yl)-4-(benzyloxy)-3-hydroxytetrahydrofuran-2-yl)methyl (*L*-phenylalanyl)sulfamate (5)

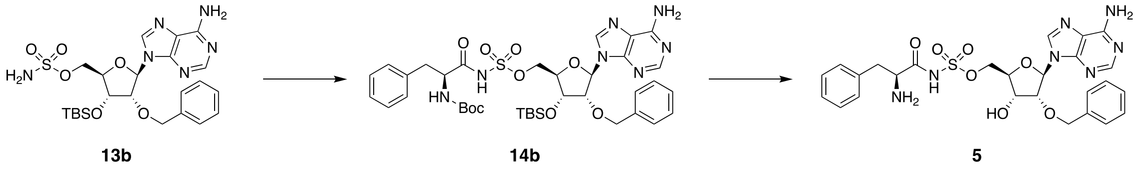

Boc-Phe-OSu (30 mg, 0.08 mmol) and Cs_2CO_3 (49 mg, 0.15 mmol) were added to a solution of compound **13b** (30 mg, 0.05 mmol) in DMF (1.0 mL). The solution was stirred at room temperature for 1 h. The reaction mixture was diluted with EtOAc. The mixture was washed with 5% citric acid, 5% $NaHCO_3$, and brine. The organic layer was dried over Na_2SO_4 and evaporated to dryness. The residue was used to next step without further purifications. Crude compound **14b** was dissolved in a 4:1 (v/v) mixture of TFA and H_2O at room temperature. After 6 h, the flask

was placed on the rotary evaporator and the TFA and H_2O were removed at reduced pressure. The residue was purified by flash chromatography (10:1 to 5:1 $CHCl_3$/MeOH) to afford compound **5** as a white solid (30 mg, 94%). ^{1}H NMR (500 MHz, Methanol-d_4) δ 8.39 (s, 1H), 8.20 (s, 1H), 7.31 – 7.26 (m, 2H), 7.26 – 7.18 (m, 4H), 7.18 – 7.06 (m, 4H), 6.16 (d, *J* = 4.8 Hz, 1H), 4.77 (d, *J* = 12.2 Hz, 1H), 4.60 (d, *J* = 12.2 Hz, 1H), 4.48 (t, *J* = 4.9 Hz, 1H), 4.43 (t, *J* = 4.6 Hz, 1H), 4.41 – 4.28 (m, 3H), 3.96 (dd, *J* = 7.9, 5.1 Hz, 1H), 3.31 – 3.27 (m, 1H), 3.08 (dd, *J* = 14.4, 7.9 Hz, 1H). ^{13}C NMR (126 MHz, Methanol-d_4) δ 175.1, 155.2, 150.9, 150.0, 141.9, 138.8, 136.3, 130.7, 129.9, 129.2, 129.1, 128.8, 128.4, 120.0, 88.1, 84.5, 82.7, 73.6, 70.7, 69.0, 58.1, 38.4. HRMS (ESI−): $[M-H]^-$ calcd for $C_{26}H_{28}N_7O_7S$, 582.1771; found, 582.1749.

((2*R*,3*R*,4*R*,5*R*)-5-(6-Amino-9*H*-purin-9-yl)-4-(cyanomethoxy)-3-hydroxytetrahydrofuran-2-yl)methyl (*L*-phenylalanyl)sulfamate (7)

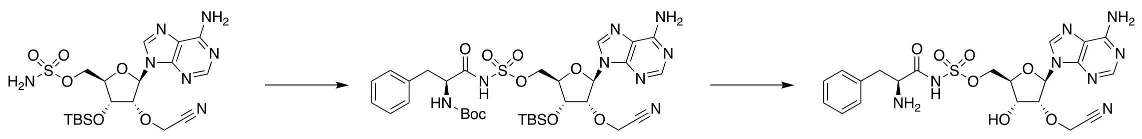

13d **14d** **7**

Boc-Phe-OSu (33 mg, 0.09 mmol) and Cs_2CO_3 (59 mg, 0.18 mmol) were added to a solution of compound **13d** (30 mg, 0.06 mmol) in DMF (1.0 mL). The solution was stirred at room temperature for 1 h. The reaction mixture was diluted with EtOAc. The mixture was washed with 5% citric acid, 5% $NaHCO_3$, and brine. The organic layer was dried over Na_2SO_4 and evaporated to dryness. The residue was used to next step without further purifications. Crude compound **14d** was dissolved in a 4:1 (v/v) mixture of TFA and H_2O at room temperature. After 6 h, the flask was placed on the rotary evaporator and the TFA and H_2O were removed at reduced pressure. The residue was purified by flash chromatography (10:1 to 5:1 $CHCl_3$/MeOH) to afford compound **7** as a white solid (28 mg, 88%). ^{1}H NMR (500 MHz, Methanol-d_4) δ 8.55 (s, 1H), 8.26 (s, 1H), 7.33 – 7.25 (m, 4H), 7.23 – 7.15 (m, 1H), 6.22 (d, *J* = 4.6 Hz, 1H), 4.69 – 4.65 (m, 1H), 4.61 (d, *J* = 8.0 Hz, 2H), 4.54 (t, *J* = 4.7 Hz, 1H), 4.37 (dd, *J* = 12.0, 3.4 Hz, 1H), 4.33 – 4.28 (m, 2H), 3.92 (dd, *J* = 8.3, 4.8 Hz, 1H), 3.05 (dd, *J* = 14.5, 8.3 Hz, 1H). ^{13}C NMR (125 MHz, Methanol-d_4) δ 175.0, 155.8, 151.8, 150.4, 141.7, 136.5, 130.6, 130.0, 128.5, 120.2, 117.4, 87.7, 84.4, 84.2, 70.6, 68.5, 58.2, 57.1, 38.5. HRMS (ESI−): $[M-H]^-$ calcd for $C_{21}H_{23}N_8O_7S$, 531.1410; found, 531.1437

Chemical biology procedures

Protein expression and purification: Recombinant protein *holo*-GrsA were expressed and purified as previously described.[4,5] This protein was overproduced and isolated as a C-terminal His-tagged construct using the *Escherichia coli* overexpression strain, BL21 (DE3), kindly provided by Prof. Mohamed A. Marahiel at Philipps-Universität Marburg, Germany.

Preparation of proteomes for in vitro labeling experiments: *Aneurinibacillus migulanus* ATCC 9999 was cultured and the whole cell lysate was isolated as described previously.[4]

Determination of K_d values of inhibitors by the competitive ELISA assay[2]: Prior to carrying out the competitive ELISA, the optimum enzyme concentration and the incubation time on streptavidin high binding capacity Coated 96-well plates (Thermo Fisher Scientific K.K.) (precoated with 1.5 μg/mL of L-Phe-AMS-biotin, structure are shown in Figure S2) were determined to establish a reproducible titration curve. In our experiments, the final concentration and the incubation time were typically 0.875 μg/mL (6.89 nM) of GrsA and 15–20 min.[2] Competitive ELISA experiments were conducted as follows: a 60-μL solution of each inhibitor in 20 mM Tris (pH 8.0), 1 mM $MgCl_2$, 1 mM TCEP and 0.0025% NP- 40 was serially diluted 2-fold across a 96-well flat-bottom plate (Corning) containing 60 μL of buffer per well. To each well, 60 μL of enzyme solution (final concentration was preadjusted) was added to each well, and the mixture was equilibrated for 1 h at room temperature. Determination of the K_d values of inhibitors **4**, **5**, and **7** for GrsA was conducted as follows. Final reaction concentrations: GrsA (0.875 μg/mL, 6.89 nM) and **4**, **5**, or **7** (6.1 nM to 100 μM). Control samples (DMSO alone) were incubated under the same conditions. In all the experiments, the total DMSO concentration was maintained at or below 2.0%. The resulting enzyme-inhibitor solutions (100 μL) from each well were transferred to wells treated with probes and incubated for 15–20 min. After extensive washing with 200 μL of PBST, wells were treated with a solution of 100 μL of an anti-6× His, monoclonal antibody (9C11, Wako Pure Chemical Industries, Ltd.) (1:5000 in PBST) for 1 h at room temperature. After three washes with 200 μL of PBST, a solution of 100 μL of goat anti-mouse-HRP conjugate (Bio-Rad Laboratories, Inc.) (1:5000 in PBST), was incubated for 1 h at room temperature, followed by three washes with 200 μL of PBST, and each well was then treated with 100 μL of 0.4 mg/mL *o*-phenylenediamine (OPD) in 0.05 M phosphate- citrate (pH 5.0) containing 0.4 mg/mL urea hydrogen peroxide at room temperature. The yellow color was allowed to develop for approximately 5 min and the reaction was quenched by the addition of 50 μL of 1 M H_2SO_4. The absorbance at 492 nm was measured using an EnVision Multilabel Reader (PerkinElmer). The results were analyzed using a Klotz plot (a double reciprocal plot of the inhibition concentration vs. the ratio of inhibitor-bound enzyme to the total added enzyme) and the slope of the line yielding the K_d for the inhibitor. All assays were performed in duplicates.

Competitive labeling of recombinant GrsA by probe 3: In a manner similar to leterature[10], recombinant GrsA (1 µM) was incubated with inhibitors **1**, **2**, or **4–9** or DMSO for 10 min at room temperature in 20 mM Tris (pH 8.0) 1 mM $MgCl_2$, 1 mM TCEP, and 0.0025% NP-40 in a 96-well plate. The mixtures were treated with probe **3** (1 µM from a 100 µM stock in DMSO). After 10 min at room temperature, the plate was placed on ice and exposed to 365 nm light for 30 min using a UV lamp (8 W) (UVP Crosslinker, Analytik Jena) at 1 cm distance from the top of the plate (without the lid). In all experiments, the total DMSO concentration was kept at 2.2%. To initiate the Click reaction, TAMRA-N_3, TCEP, TBTA, and $CuSO_4$ were added in the final concentrations of 100 µM, 1 mM, 100 mM, and 1 mM, respectively. After 1 h at room temperature, 5× SDS-loading buffer (strong reducing) was added, and the samples were heated at 95 °C for 5 min. Samples were then separated by 1D SDS-PAGE and fluorescence gel visualization was performed using a Typhoon 9410 Gel and Blot Imager (λ_{ex} = 532 nm and λ_{em} = 580 nm) (GE Healthcare).

In vitro labeling of endogenous GrsA: In a manner similar to leterature[10], *A. migulanus* ATCC 9999 proteome (2 mg/mL) was treated with probe **3** (1 µM from a 100 µM stock in DMSO) in 20 mM Tris (pH 8.0), 1 mM $MgCl_2$, 1 mM TCEP, 0.05% Igepal CA-630, 0.2 mg/mL lysozyme, and protease inhibitor cocktail in a 96-well plate. Inhibition studies were performed by pre-incubation of *A. migulanus* ATCC 9999 proteome (2.0 mg/mL) with inhibitors **1**, **2**, or **4–9** (100 µM from a 10 mM stock in DMSO) for 10 min at room temperature. In all experiments, the total DMSO concentration was kept at 2.2%. After 10 min at room temperature, the plate was placed on ice and exposed to 365 nm light for 30 min using a UV lamp (8 W) (UVP Crosslinker, Analytik Jena) at 1 cm distance from the top of the plate (without the lid), reacted with TAMRA-N_3 at room temperature, and separated by 1D SDS-PAGE. Fluorescent gel visualization was performed using a Typhoon 9410 Gel and Blot Imager (λ_{ex} = 532 nm and λ_{em} = 580 nm) (GE Healthcare).

Labeling of intracellular GrsA in living bacterial cells: In a manner similar to leterature[10], *A. migulanus* ATCC 9999 was grown at 37 °C in YPG medium. Cultures (24 h, OD_{600} = 36) were harvested, aliquoted to Eppendolf 1.5 mL tubes, washed twice with 1 mL of PBS, resuspended in 1 mL of PBS, and treated with probe **3** (1 µL, 10 µM from a 10 mM stock in DMSO) in either the absence or presence of inhibitors **1**, **2**, or **4–9** (10 or 100 µM from a 1 or 10 mM stock in DMSO). In all experiments, the DMSO concentration was maintained at a level of 0.1%. After 10 min at room temperature, the bacterial cells were harvested, washed twice with 1 mL of PBS, resuspended in 500 µL of PBS, and transferred into a 96-well plate (100 µL each). The plate was placed on ice and exposed to 365 nm light for 30 min using a UV lamp (8 W) (UVP Crosslinker, Analytik Jena) at 1 cm distance from the top of the plate (without the lid). The cells were then harvested by centrifugation and stored in a freezer until use. Proteomes (2 mg/mL) were reacted with TAMRA-N_3 for 1 h at room temperature and separated by gel electrophoresis. Fluorescence gel visualization was performed using a Typhoon 9410 Gel and Blot Imager (λ_{ex} = 532 nm and λ_{em} = 580 nm) (GE Healthcare).

References

1. Ishikawa, F.; Miyamoto, K.; Konno, S.; Kasai, S.; Kakeya, H. Accurate detection of adenylation domain functions in nonribosomal peptide synthetases by an enzyme-linked immunosorbent assay system using active site-directed probes for adenylation domains. *ACS Chem. Biol.* **2015**, 10, 2816–2826.
2. Ishikawa, F.; Kakeya, H. A competitive enzyme-linked immunosorbent assay system for adenylation domains in nonribosomal peptide synthetases. *ChemBioChem* **2016**, 17, 474–478.
3. Ishikawa, F.; Nohara, M.; Takashima, K.; Tanabe, G. Probing the compatibility of an enzyme-linked immunosorbent assay toward the reprogramming of nonribosomal peptide synthetase adenylation domains. *ChemBioChem* **2020**, 21, 3056–3061.
4. Ishikawa, F.; Kakeya, H. Specific enrichment of nonribosomal peptide synthetase module by an affinity probe for adenylation domains. *Bioorg. Med. Chem. Lett.* **2014**, 24, 865–869.
5. Konno, S.; Ishikawa, F.; Suzuki, T.; Dohmae, N.; Burkart, M. D.; Kakeya, H. Active site-directed proteomic probes for adenylation domains in nonribosomal peptide synthetases. *Chem. Commum.* **2015**, 51, 2262–2265.
6. Peterson, M. A.; Nilsson, B. L.; Sarker, S.; Doboszewski, B.; Zhang, W.; Robins, M. Amide-linked ribonucleoside dimers derived from 5'-amino-5'-deoxy- and 3'-(carboxymethyl)-3'-deoxynucleoside precursors. *J. Org. Chem.* **1999**, 64, 8183–8192.
7. Li, J.; Wei, H.; Zhou, M. Structure-guided design of a methyl donor cofactor that controls a viral histone H3 lysine 27 methyltransferase activity. *J. Med. Chem.* **2011**, 54, 7734–7738.
8. Gottlieb, H. E.; Kotlyar, V.; Nudelman, A. NMR chemical shifts of common laboratory solvents as trace impurities. *J. Org. Chem.* **1997**, 62, 7512-7515.
9. Still, W. C.; Kahn, A.; Mitra, A. Rapid chromatographic technique for preparative separations with moderate resolution. *J. Org. Chem.* **1978**, 43, 2923–2925.
10. Ishikawa, F.; Konno, S.; Uchida, C.; Suzuki, T.; Takashima, K.; Dohmae, N.; Kakeya, H.; Tanabe, G. Chemoproteomics profiling of surfactin-producing nonribosomal peptide synthetases in living bacterial cells. *Cell. Chem. Biol.* **2022**, 29, 145–156.

^{1}H NMR (500 MHz) and ^{13}C NMR (125 MHz) spectra of **10d** in methanol-d_4

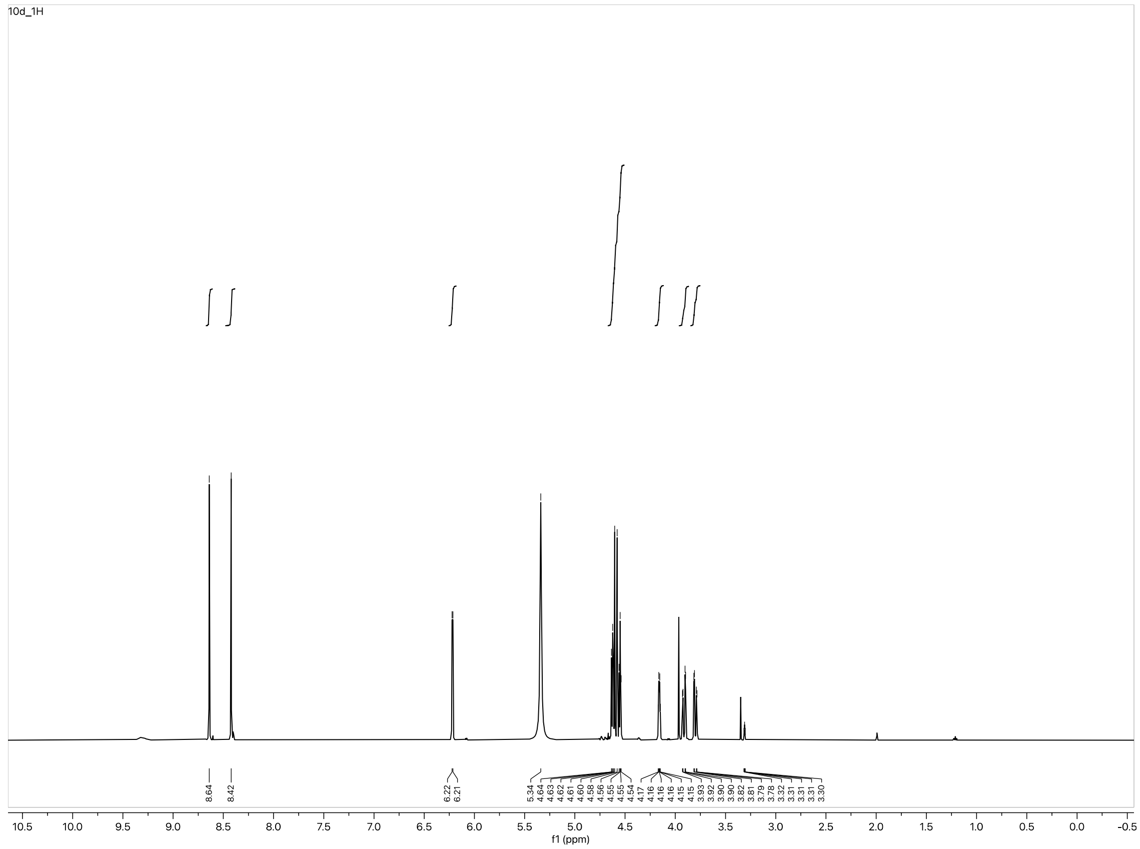

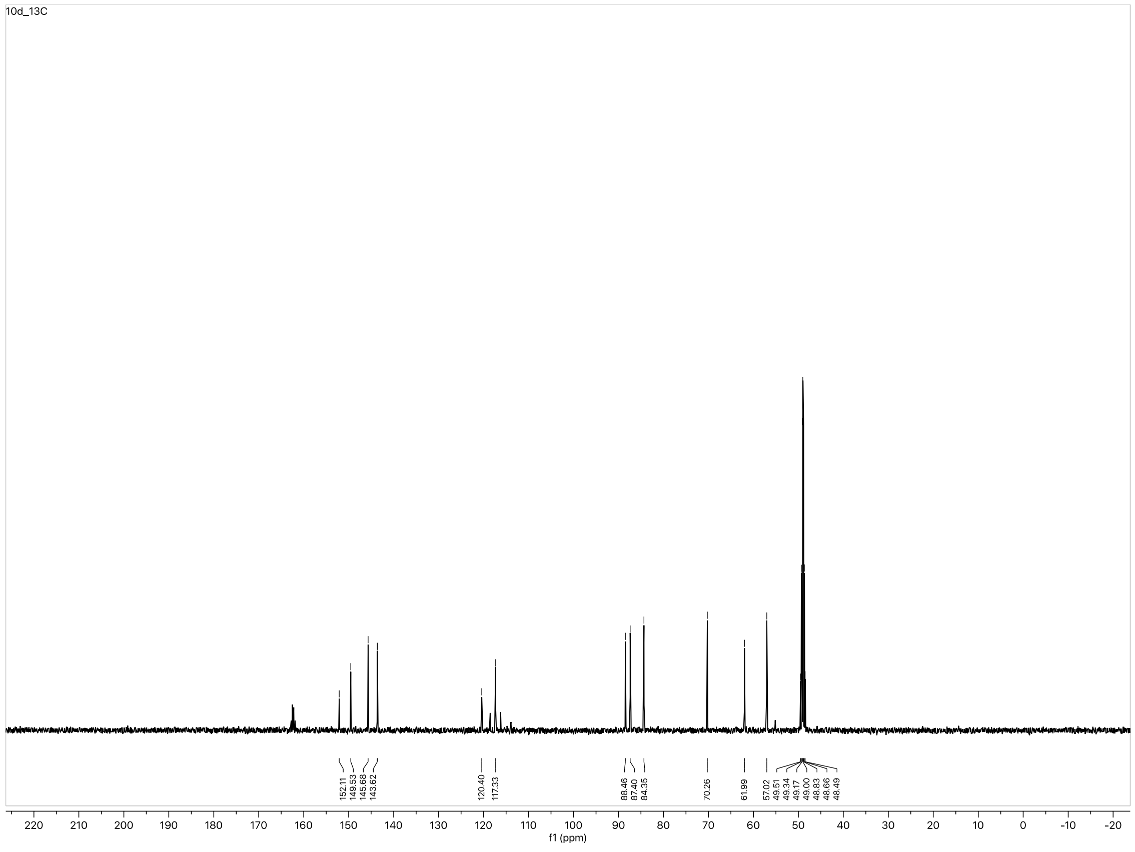

^1H NMR (500 MHz) and ^{13}C NMR (125 MHz) spectra of **11b** in $CDCl_3$

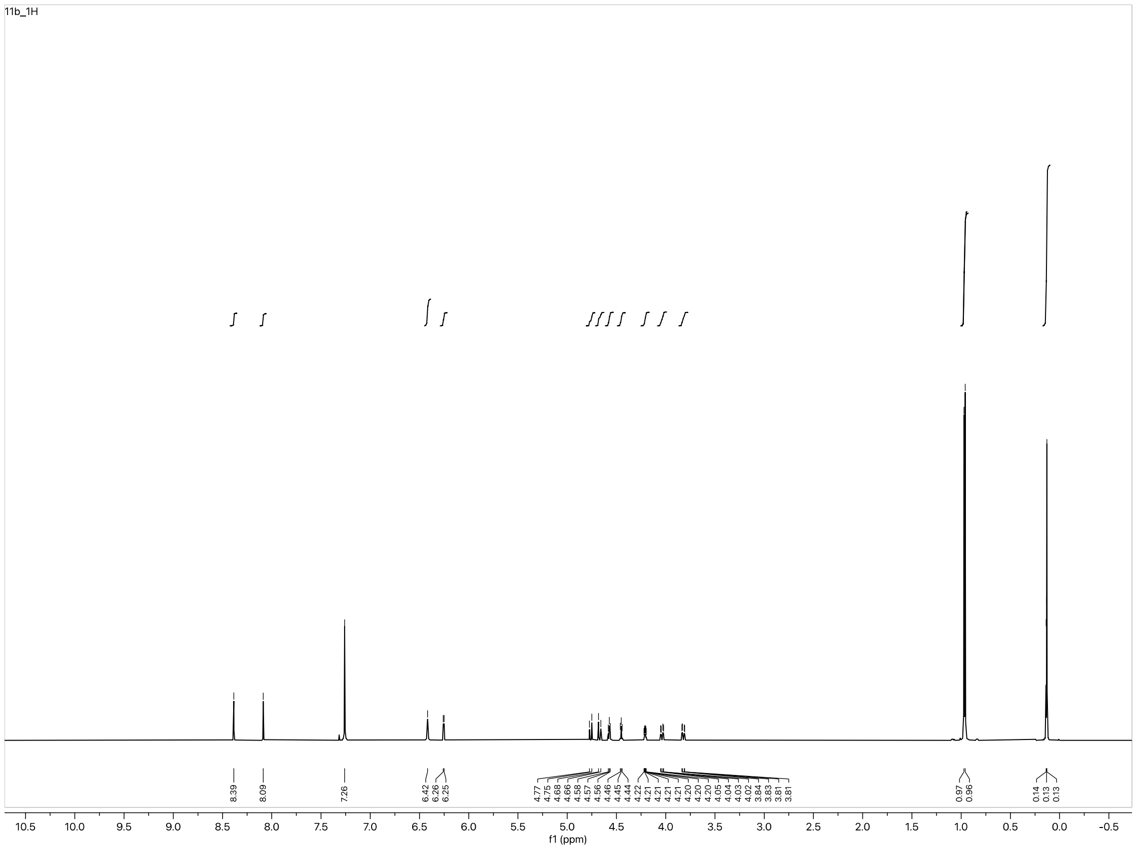

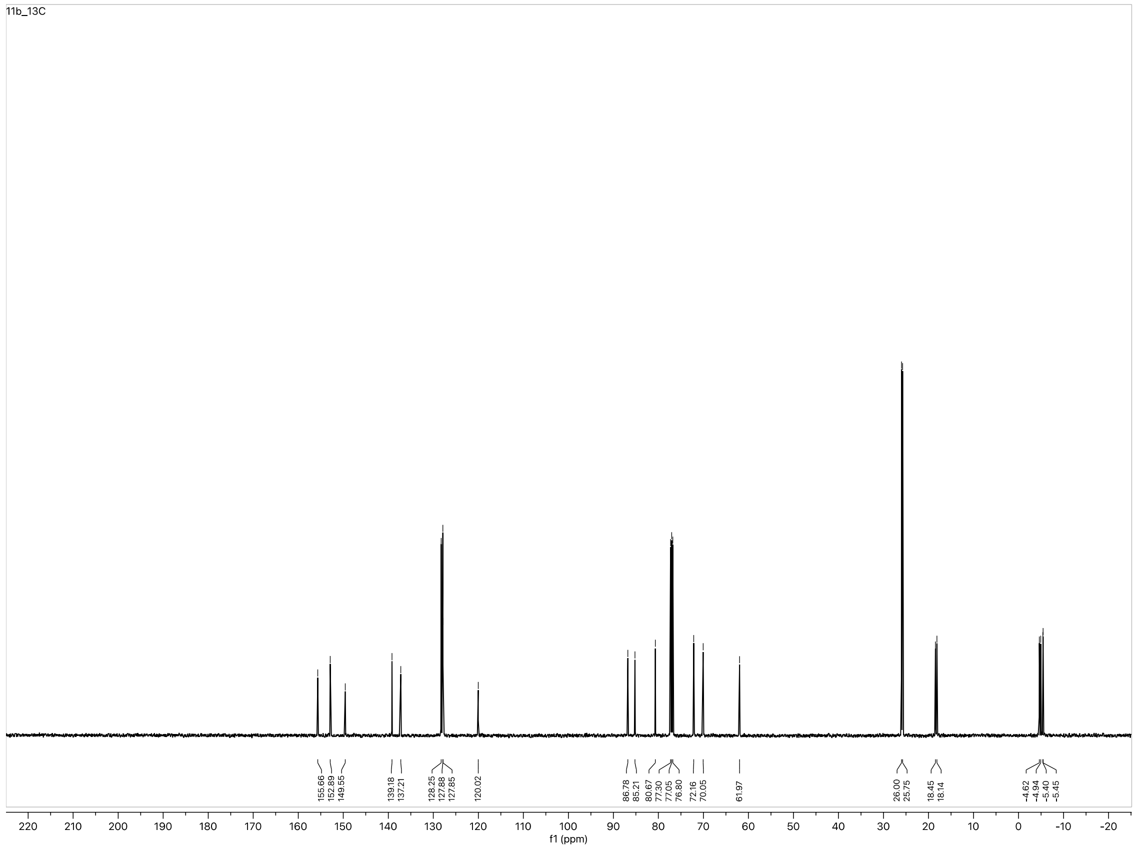

^1H NMR (500 MHz) and ^{13}C NMR (125 MHz) spectra of **11d** in $CDCl_3$

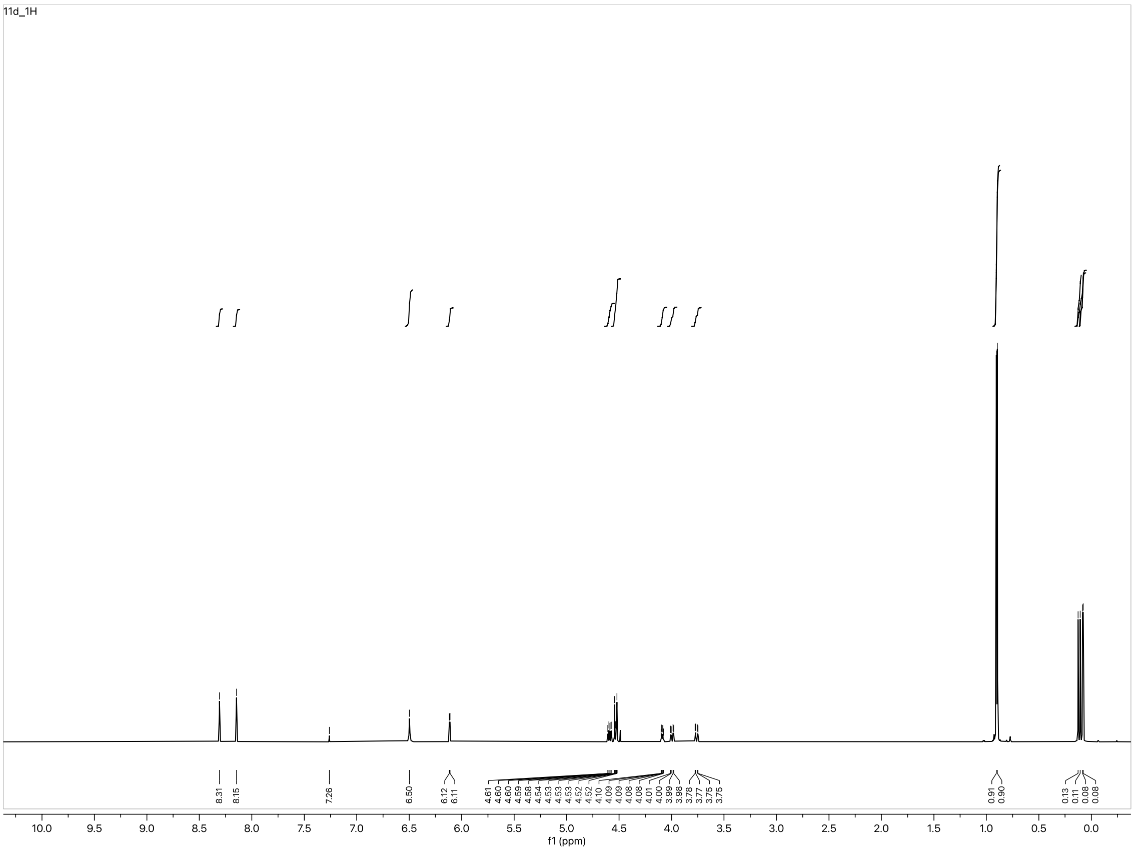

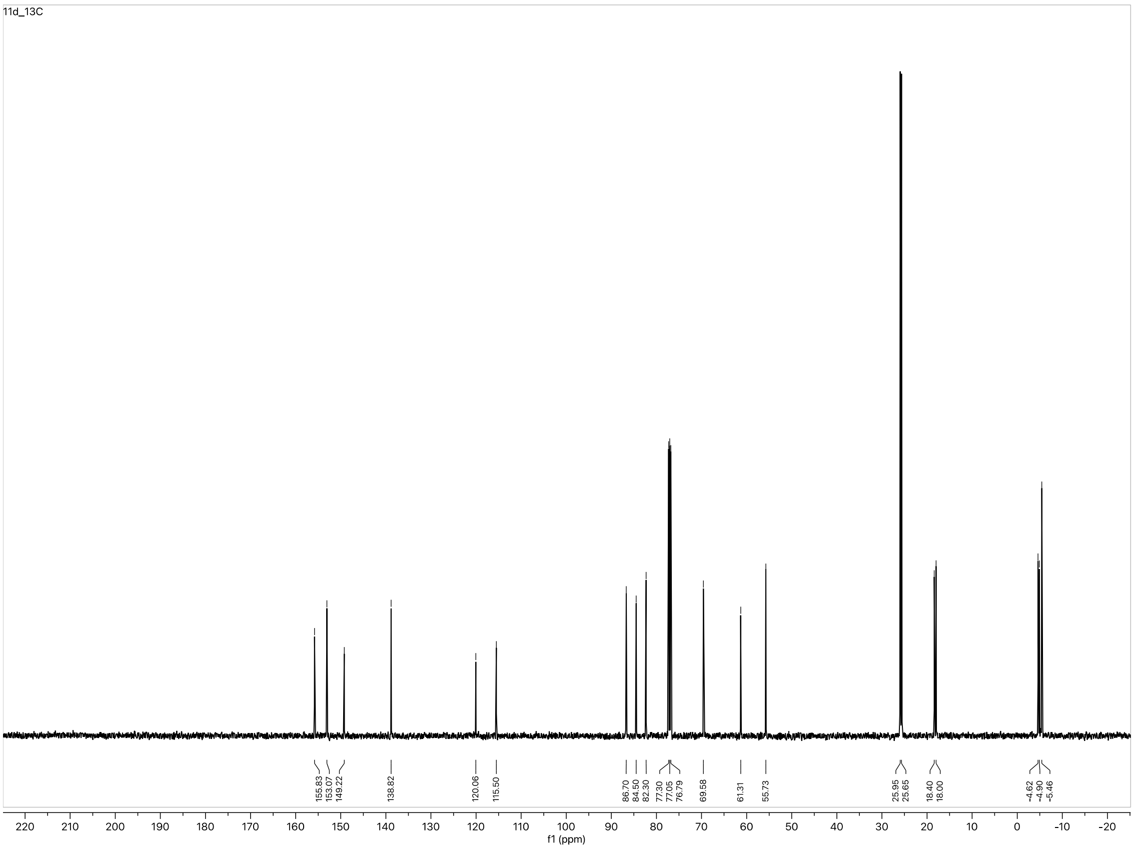

^{1}H NMR (500 MHz) and ^{13}C NMR (125 MHz) spectra of **12b** in $CDCl_3$

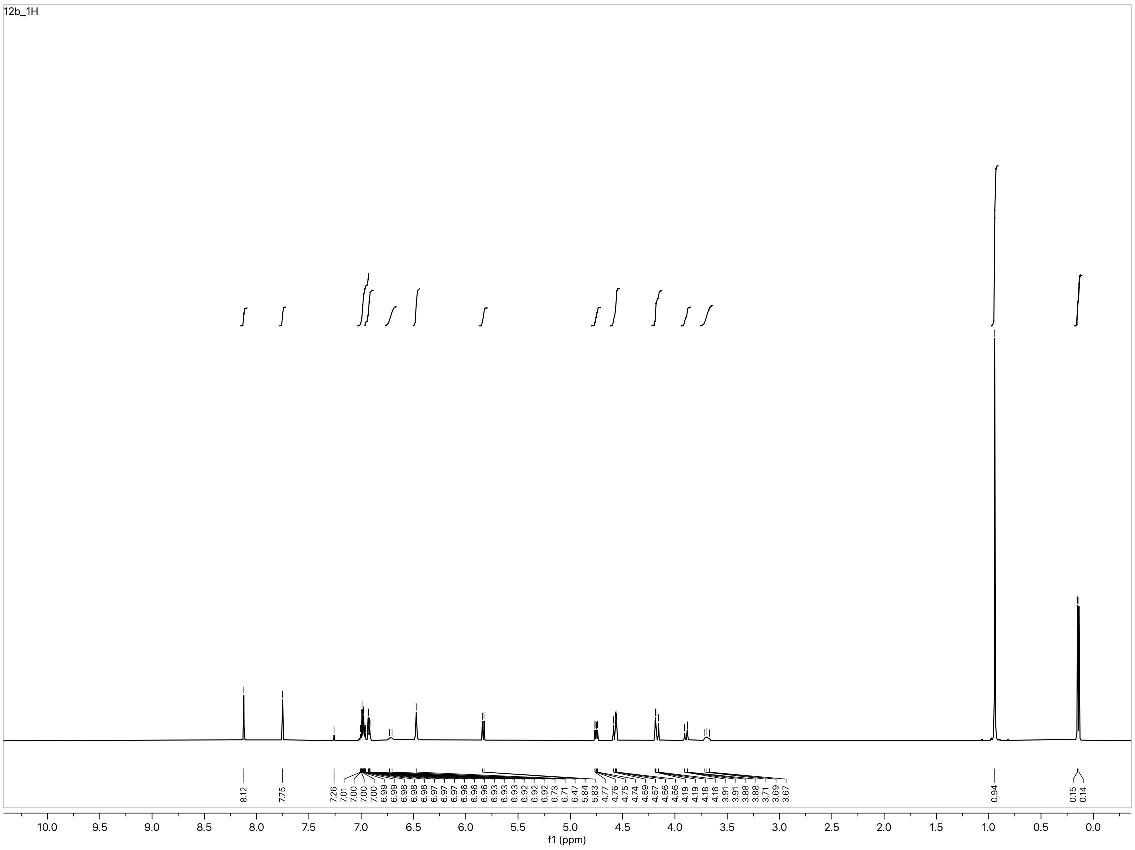

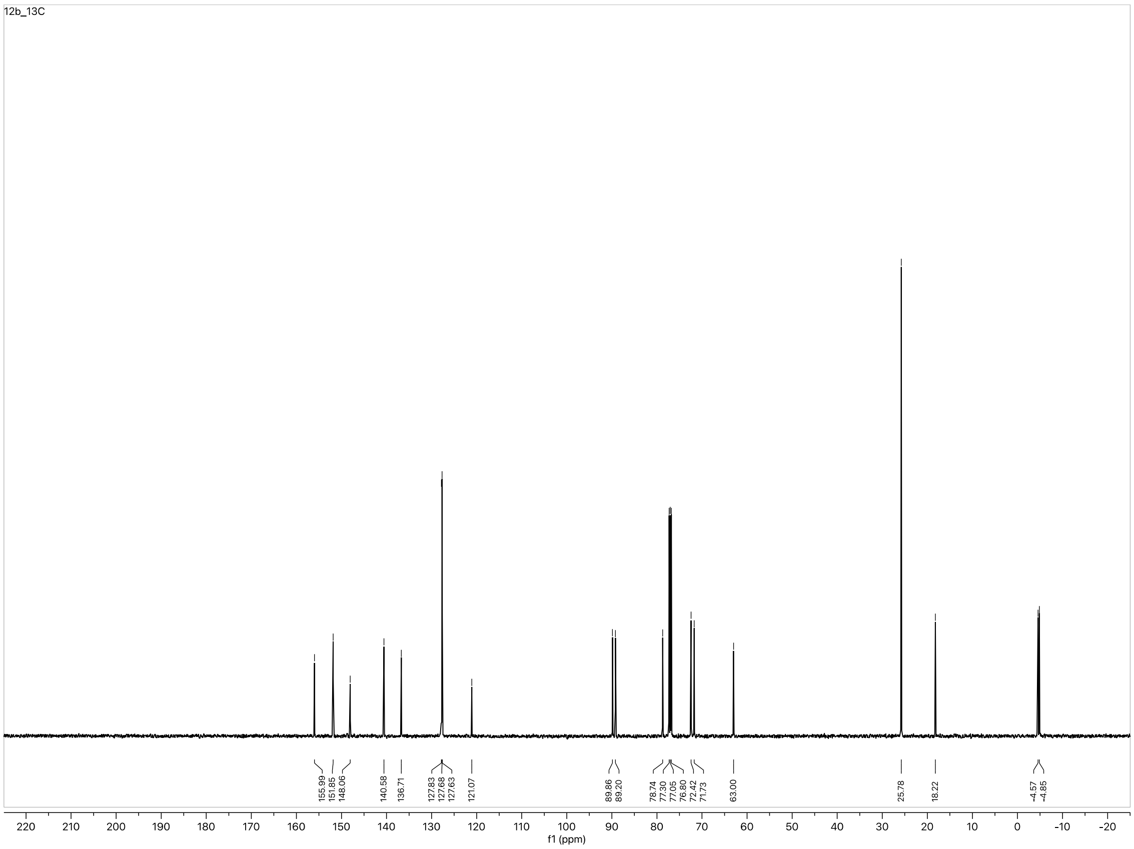

^{1}H NMR (500 MHz) and ^{13}C NMR (125 MHz) spectra of **12d** in $CDCl_3$

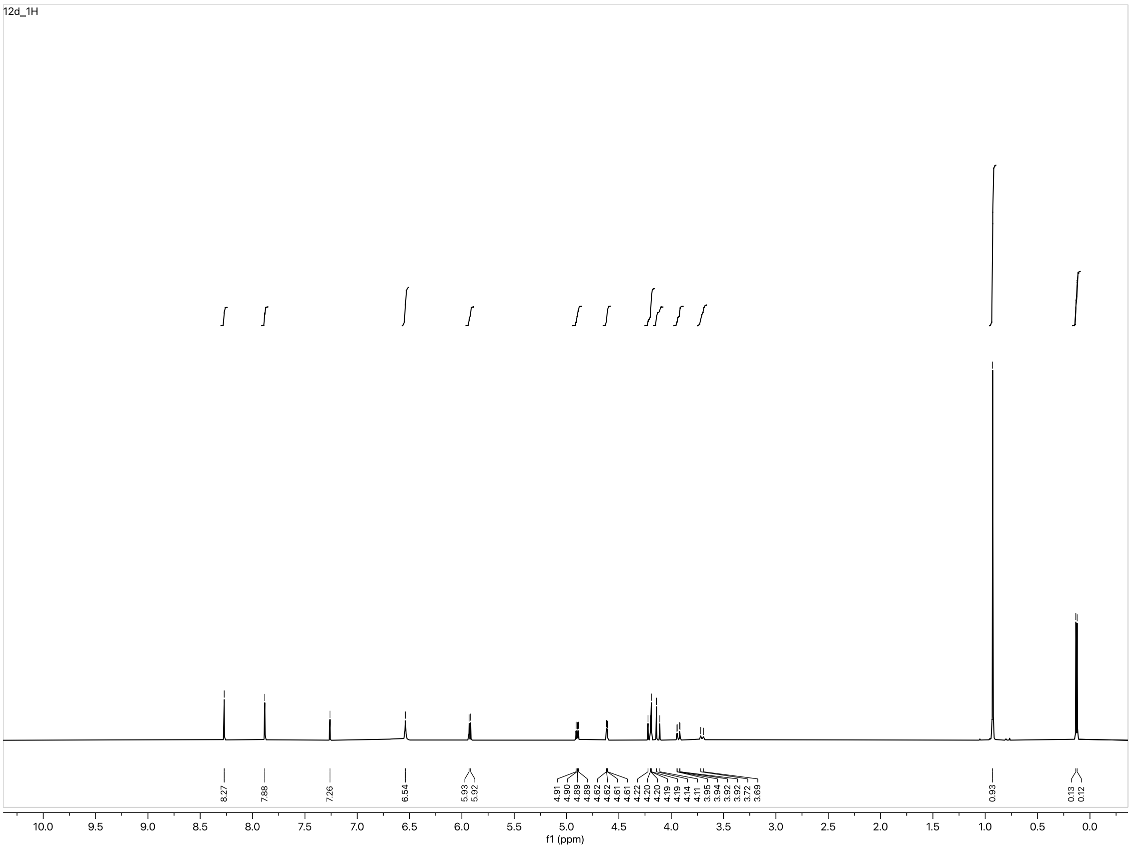

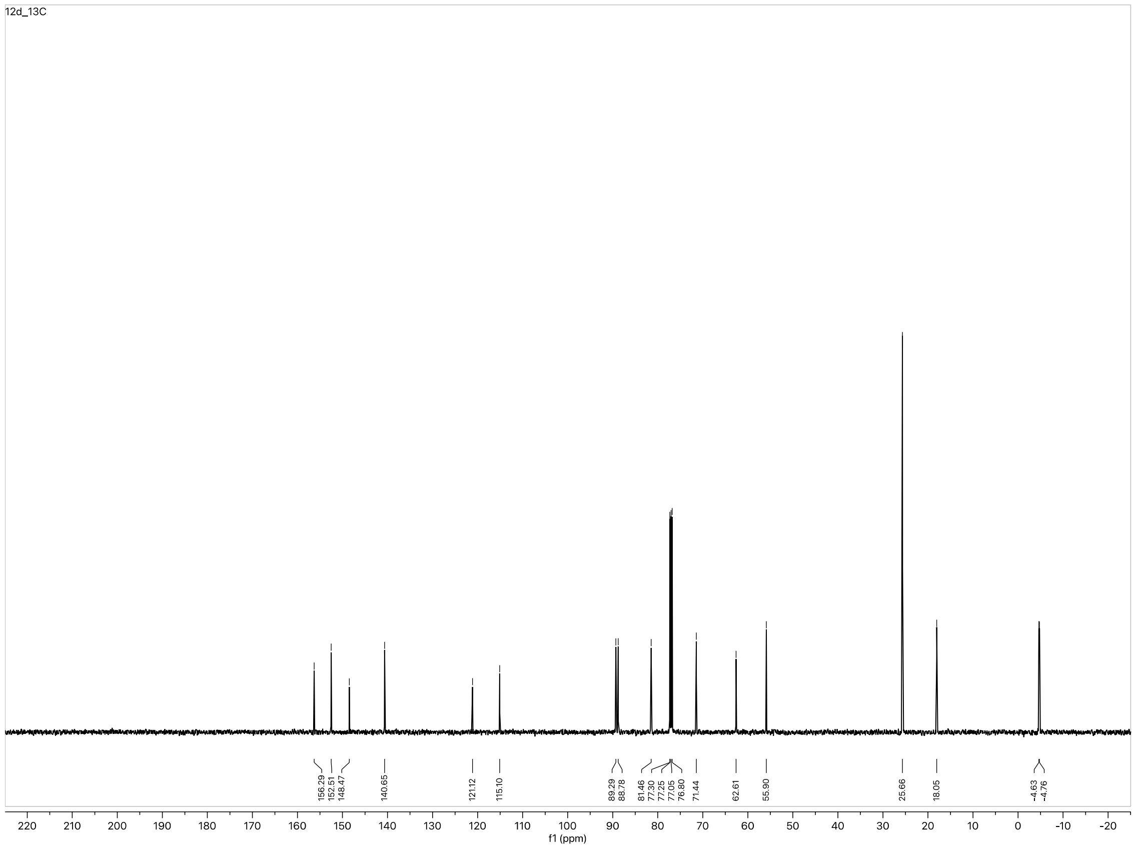

^{1}H NMR (500 MHz) and ^{13}C NMR (125 MHz) spectra of **13a** in methanol-d_4

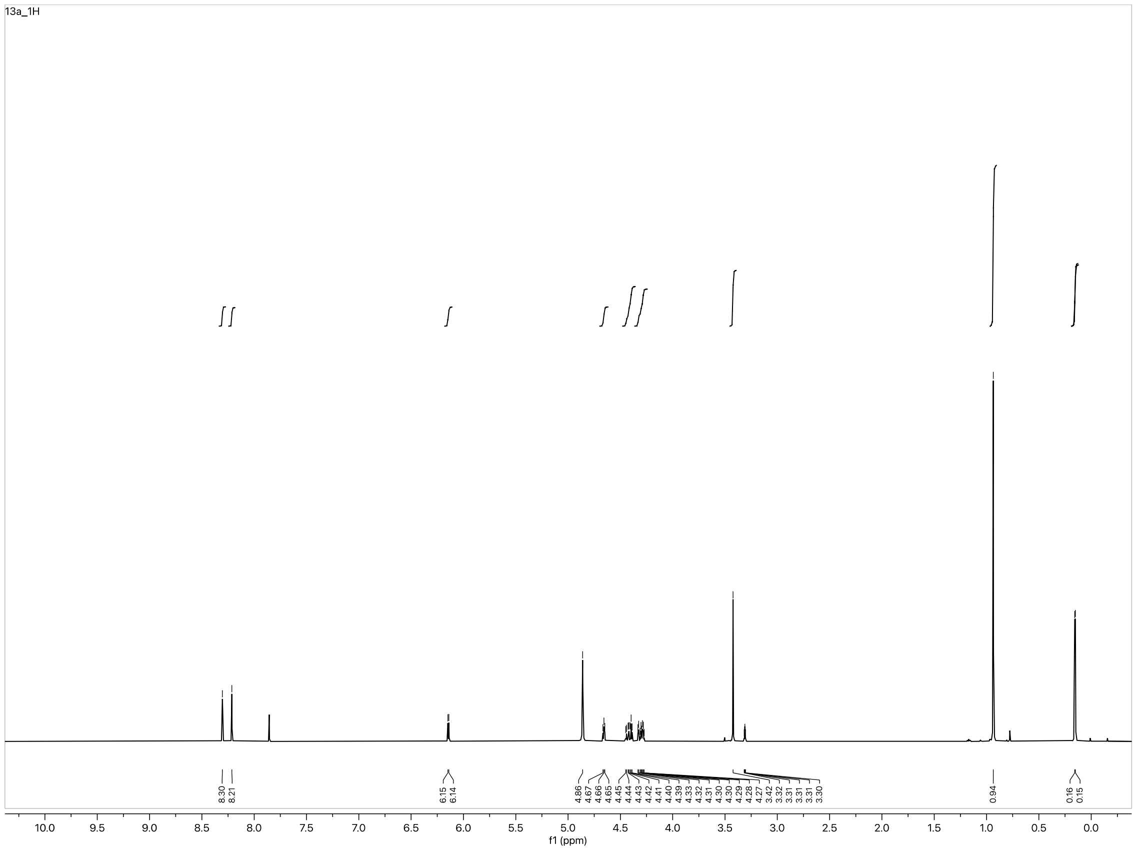

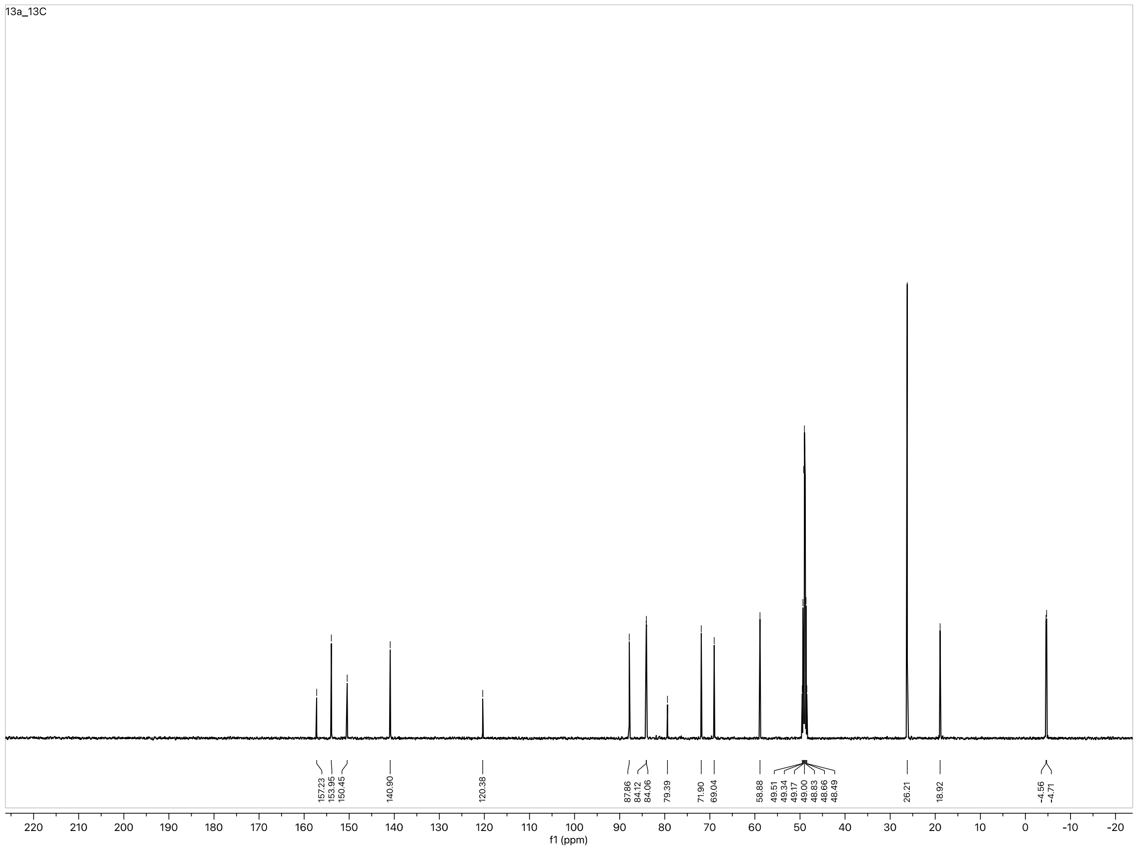

^{1}H NMR (500 MHz) and ^{13}C NMR (125 MHz) spectra of **13b** in methanol-d_4

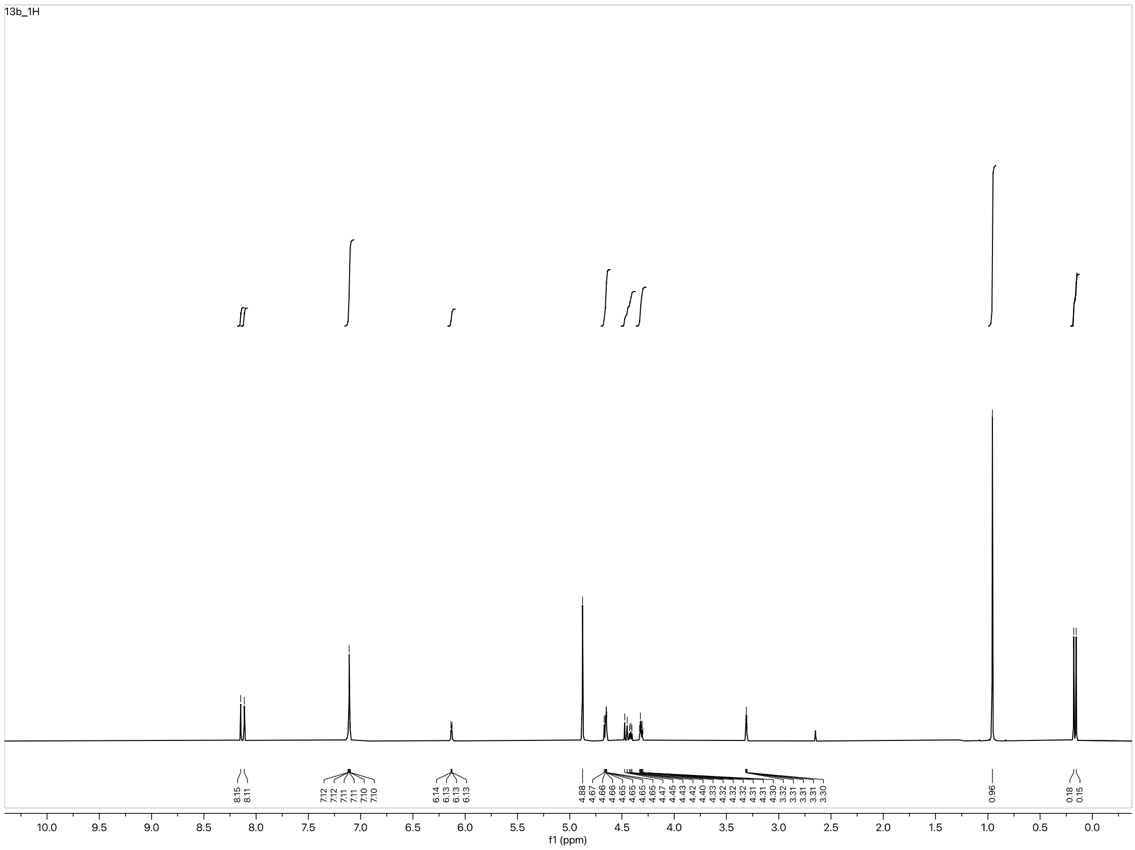

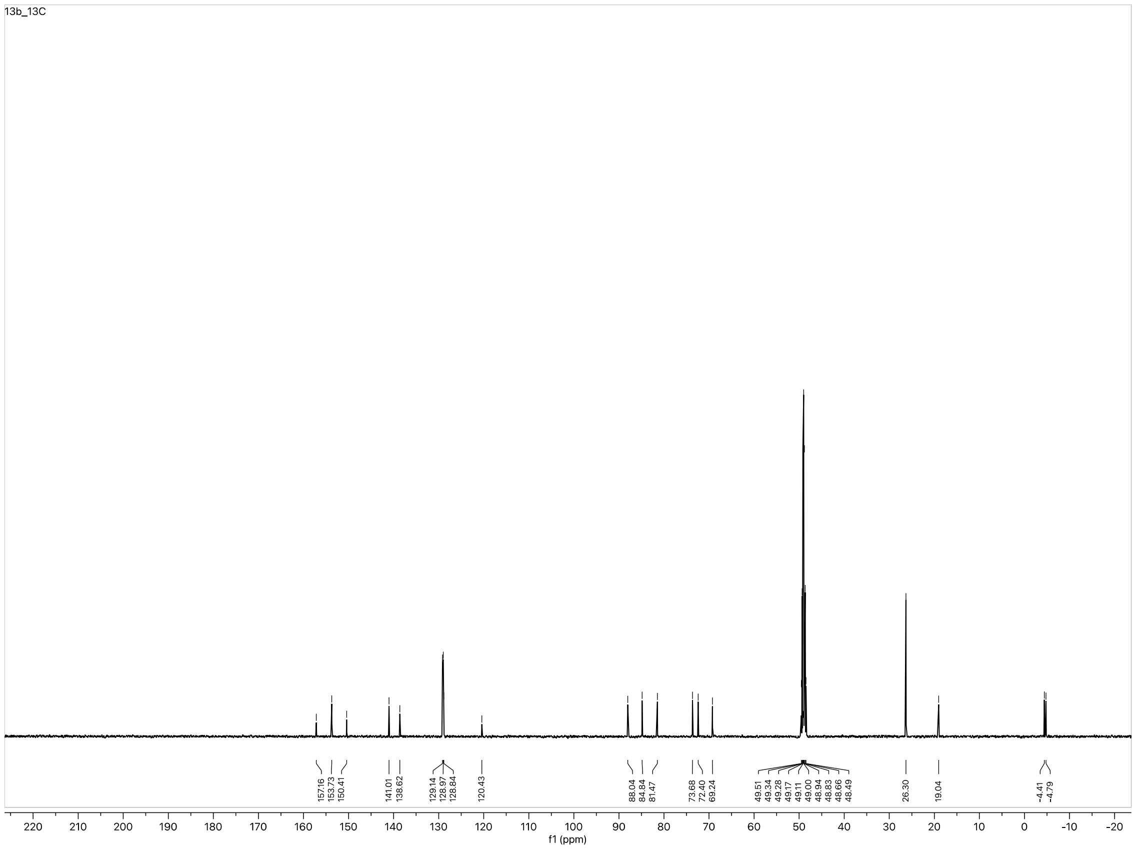

^{1}H NMR (500 MHz) and ^{13}C NMR (125 MHz) spectra of **13d** in methanol-d_4

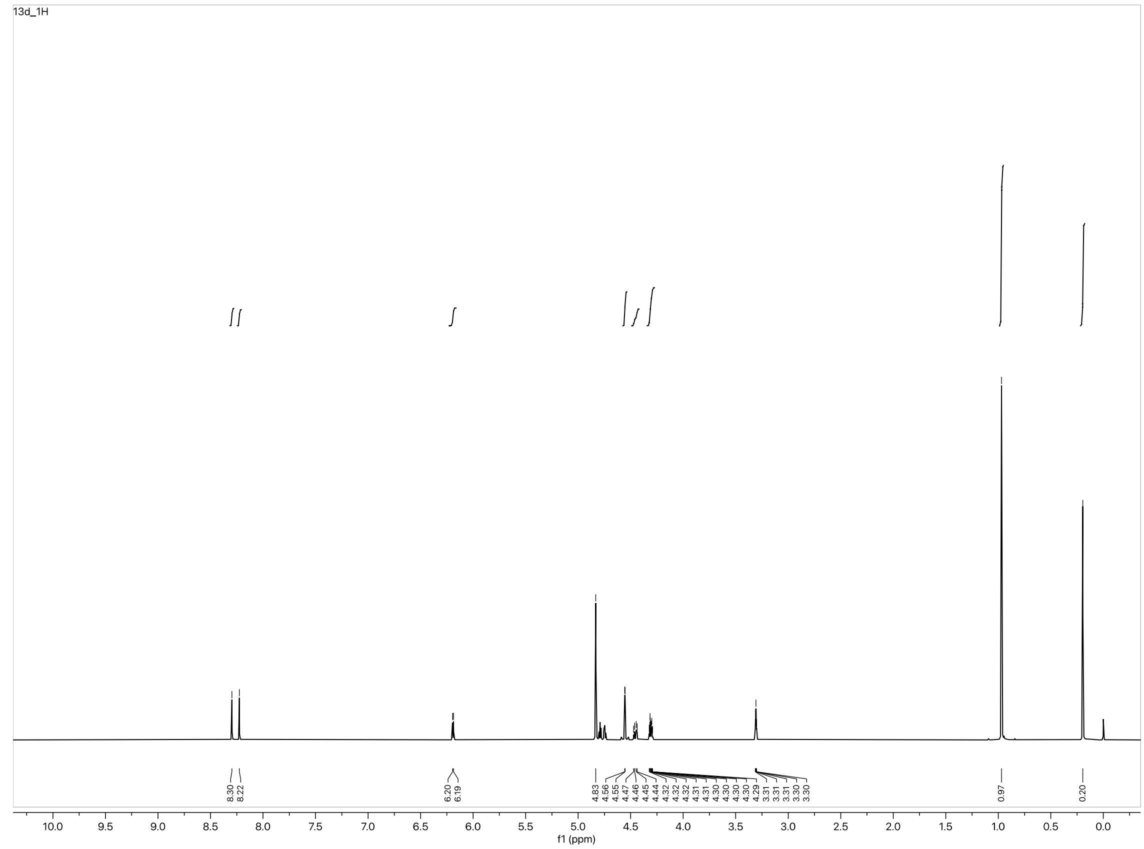

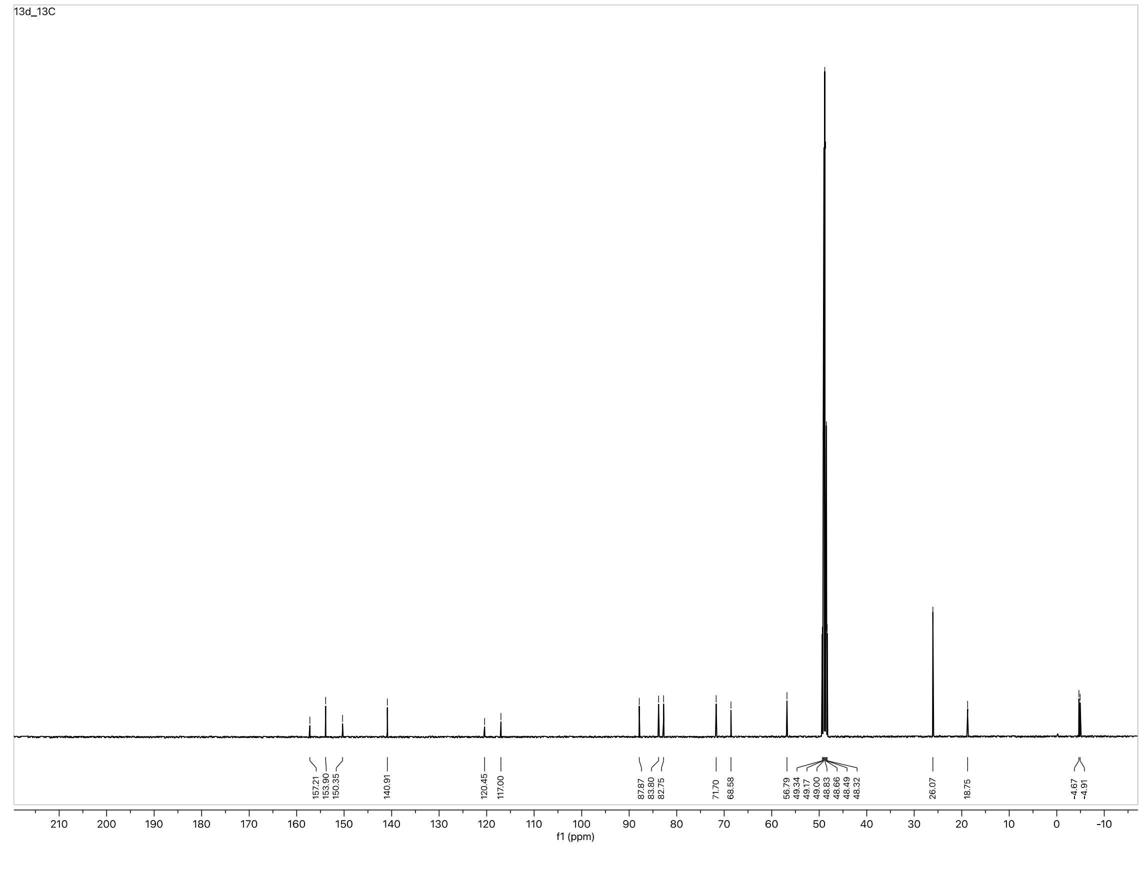

^{1}H NMR (500 MHz) and ^{13}C NMR (125 MHz) spectra of **4** in methanol-d_4

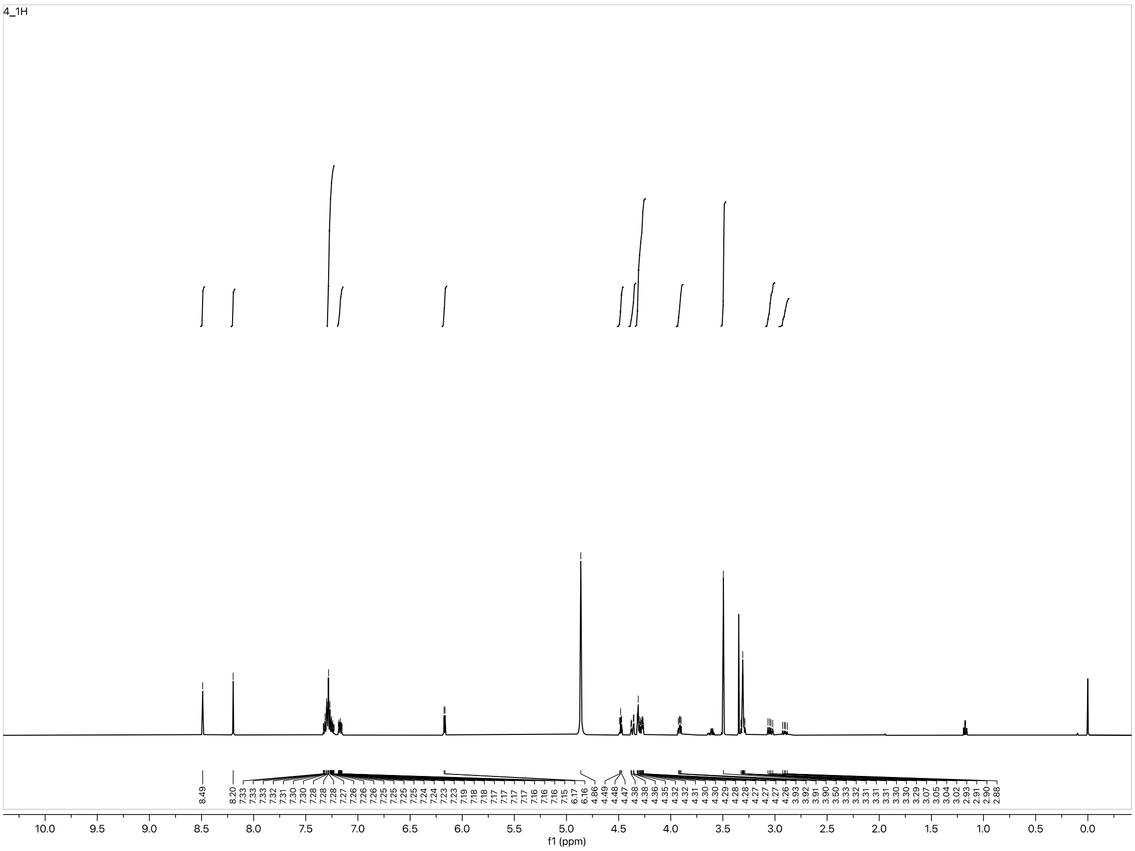

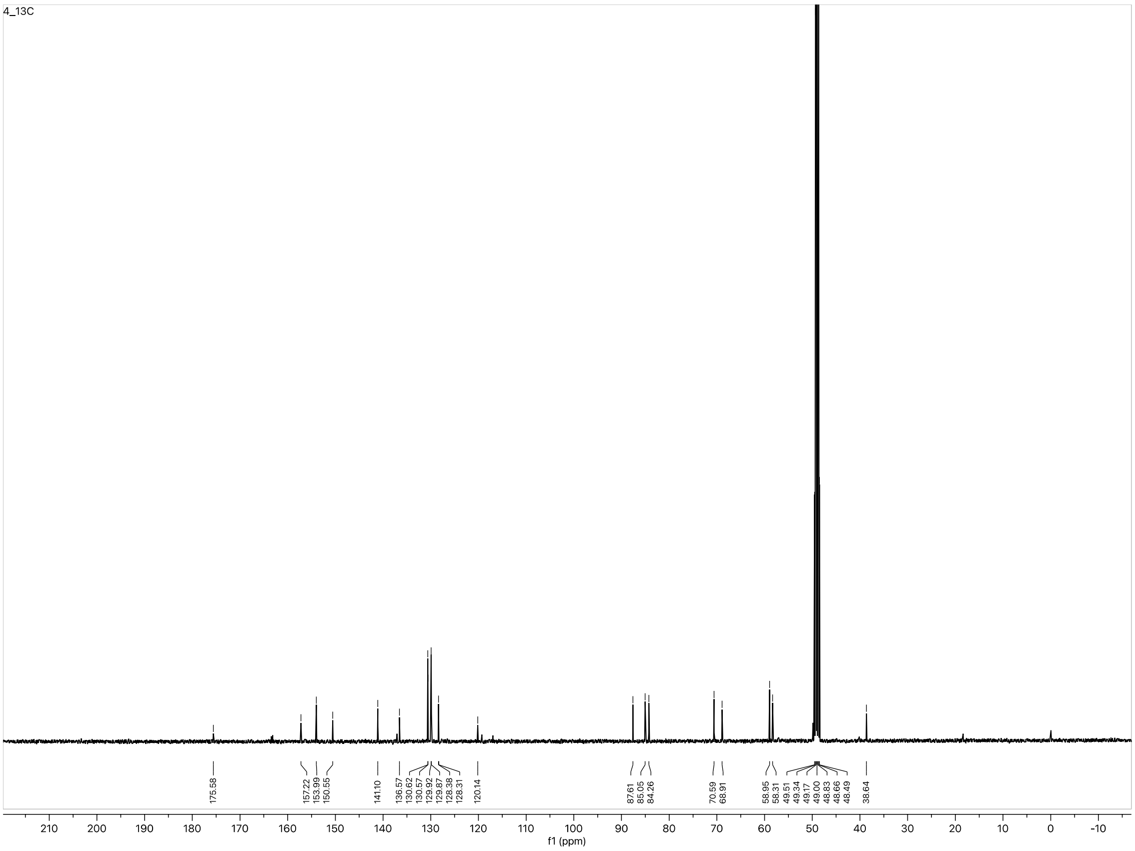

^{1}H NMR (500 MHz) and ^{13}C NMR (125 MHz) spectra of **5** in methanol-d_4

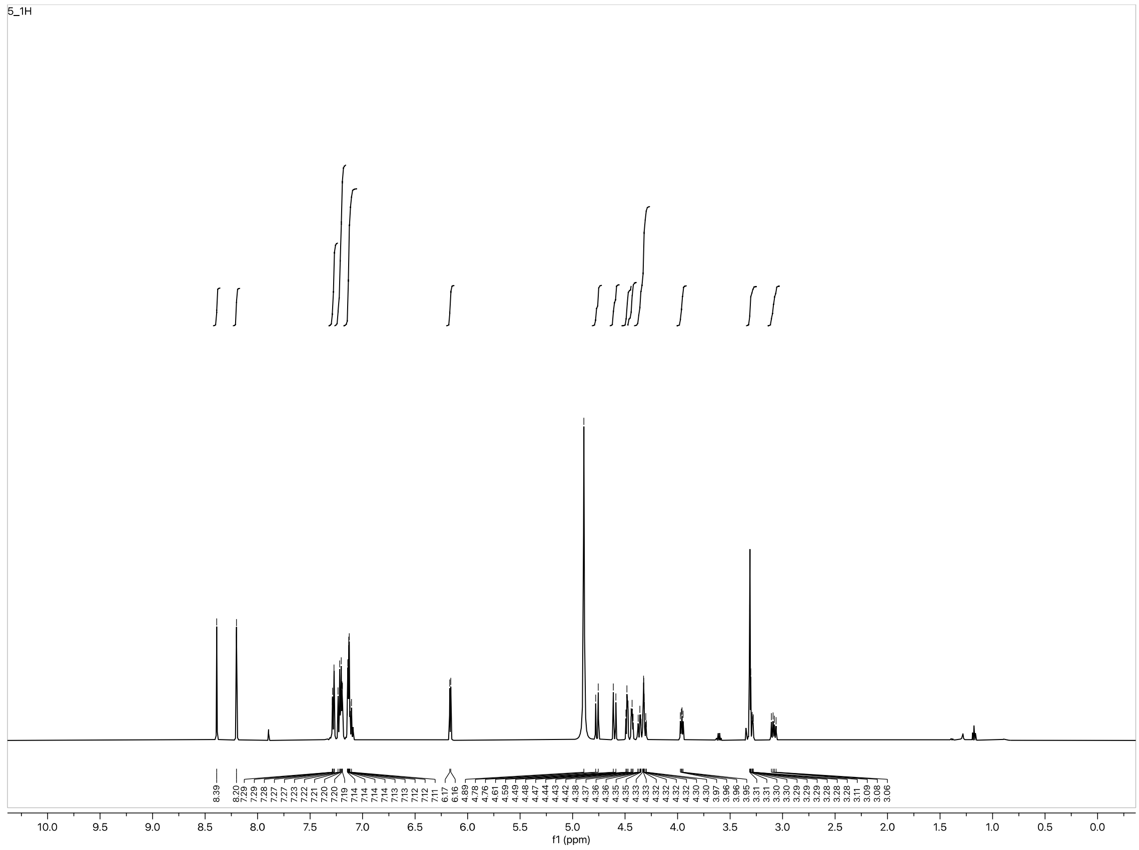

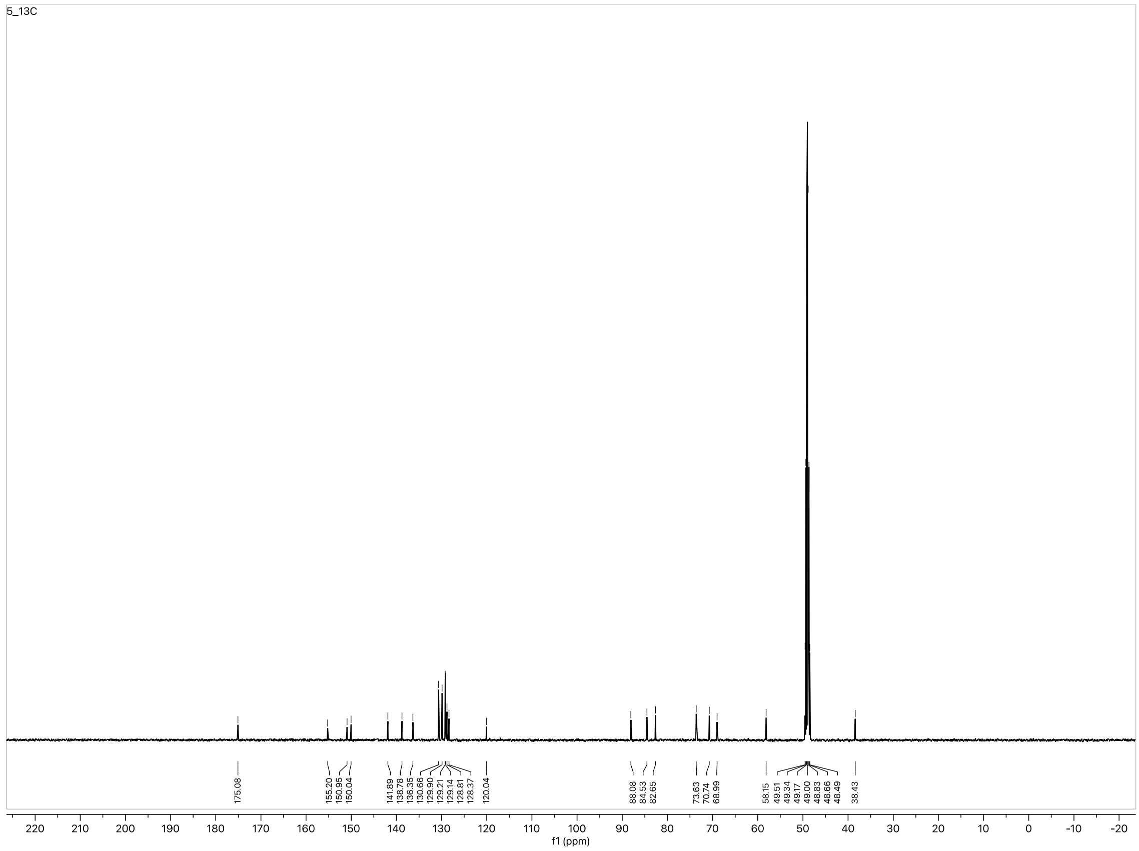

^{1}H NMR (500 MHz) and ^{13}C NMR (125 MHz) spectra of **7** in methanol-d_4

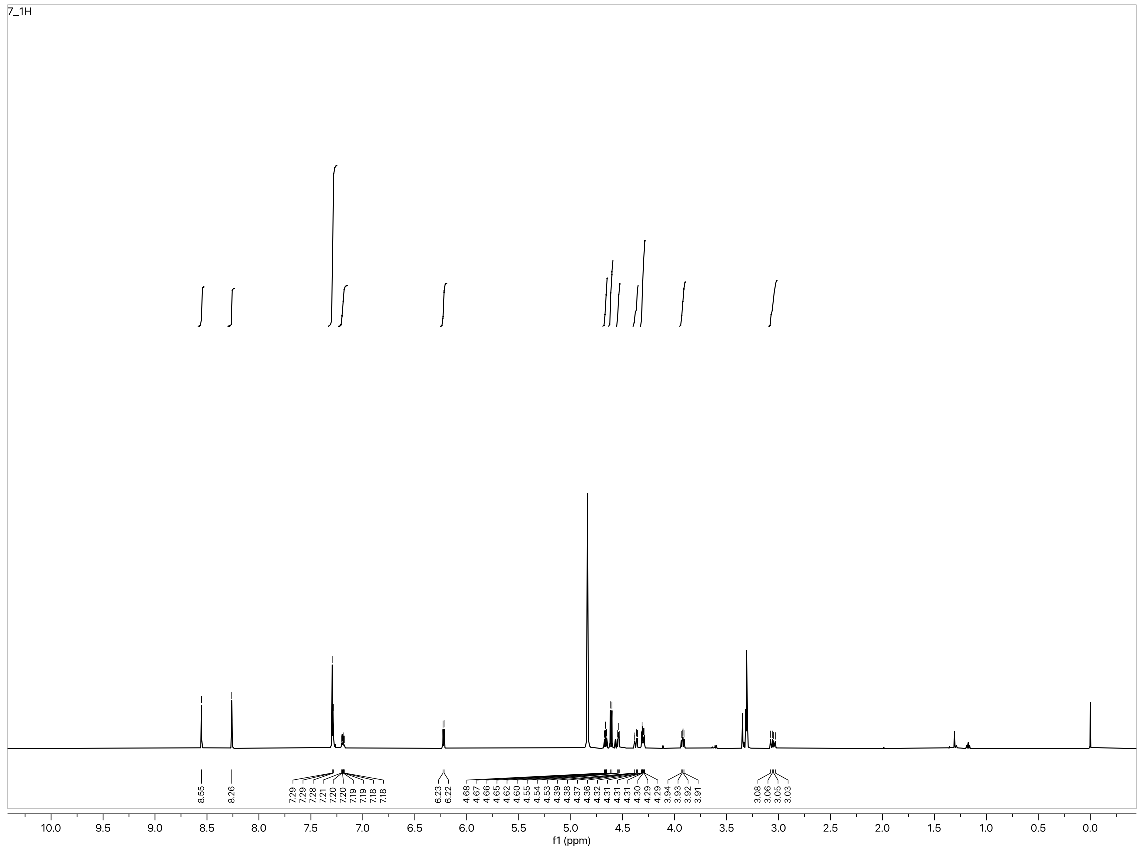

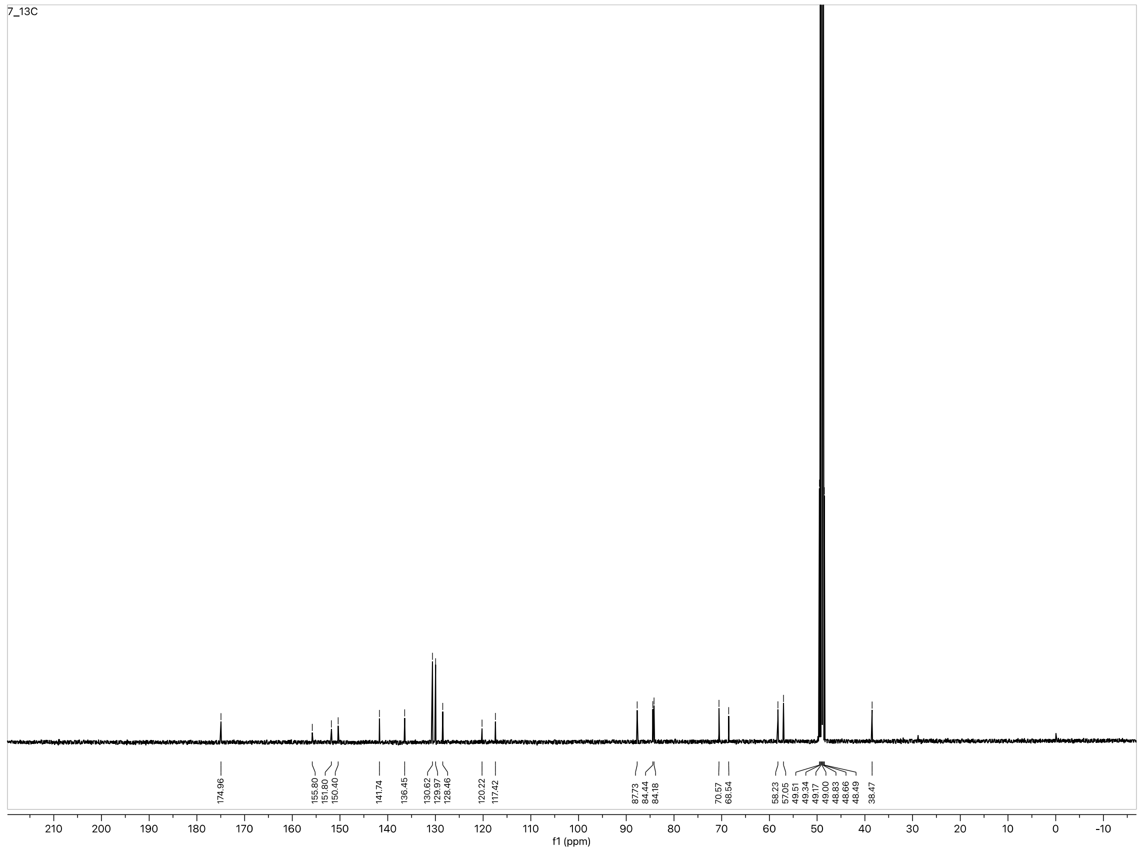

Supplement: File 1 — Additional Figures, experimental part and NMR spectra. [file Beilstein_J_Org_Chem-20-445-s001.pdf]
